# Investigation of the In Vitro and In Vivo Metabolism and μ‐Opioid Receptor Affinity of the Nitazene *N*‐Pyrrolidino Fluetonitazene

**DOI:** 10.1002/dta.70095

**Published:** 2026-06-02

**Authors:** Severin Zemp, Gaia Alluisetti, Wolfgang Weinmann, Bettina Schrag, Frank Sporkert, Maurine Leclerc, Katharina Elisabeth Grafinger

**Affiliations:** ^1^ Forensic Toxicology and Chemistry Institute of Forensic Medicine Bern, University of Bern Bern Switzerland; ^2^ Graduate School for Cellular and Biomedical Sciences University of Bern Bern Switzerland; ^3^ Department of Forensic Medicine ICH Central Institute Sion Switzerland; ^4^ Forensic Toxicology and Chemistry Unit University Centre of Legal Medicine Lausanne‐Geneva Lausanne Switzerland

## Abstract

*N*‐pyrrolidino fluetonitazene (*N*‐pyrrolidino‐4′‐(2‐fluoroethoxy) nitazene) also known as fluetonitazepyne, is the fluorinated analogue of etonitazepyne, belonging to the group of new synthetic opioids (NSOs), which are among the fastest‐growing classes of new psychoactive substances. In the present study, the *N*‐pyrrolidino fluetonitazene metabolism was investigated in vitro using pooled human liver microJsomes (pHLM) and in authentic samples from a forensic postmortem case. Qualitative analysis was performed using liquid chromatography–high‐resolution tandem mass spectrometry (LC‐HR‐MS/MS). Confirmation and semiquantitative analysis of *N*‐pyrrolidino fluetonitazene in urine and blood were carried out using liquid chromatography tandem mass spectrometry (LC–MS/MS). The μ‐opioid (MOR) receptor affinity of *N*‐pyrrolidino fluetonitazene was determined using an LC–MS/MS‐based competitive binding assay. In total, eight different metabolites for *N*‐pyrrolidino fluetonitazene were tentatively identified in vitro in pHLM incubations of which three (**M2** *O*‐dealkylation, **M6** oxidative deamination and **M8** carboxylation to *N*‐butanoic acid) were also found in the postmortem urine sample. In the blood samples, only one metabolite was found (**M9** formed by *N*‐acetylation of 5‐aminofluetodesnitazene). This metabolite was only observed in the in vivo samples and was the most abundant metabolite in blood and urine samples. We also detected metabolite **M2** (formed by 4′‐hydroxylation), which is common in the metabolism of other nitazepyne‐type substances. For the confirmation of *N*‐pyrrolidino fluetonitazene, we recommend including **M8** and **M9** as analytical target compounds, since they are specific *N*‐pyrrolidino fluetonitazene metabolites. Furthermore, we demonstrated that the LC–MS/MS‐based MOR receptor affinity assay yields valid results for *N*‐pyrrolidino fluetonitazene that are consistent with those obtained using established radioligand‐based methods.

## Graphical abstract

Eight metabolites for *N*‐pyrrolidino fluetonitazene were identified in vitro, three of which (**M2, M6** and **M8**) were present in an authentic urine sample. **M2** was the most abundant in vivo metabolite and is a common marker metabolite of nitazepyne‐type substances. The LC–MS/MS MOR affinity assay produced results consistent with radioligand methods.

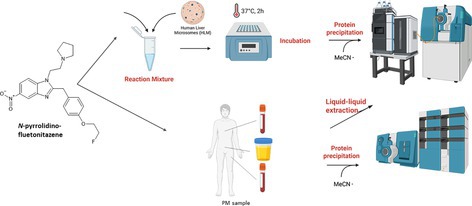

## 1 Introduction

‘Nitazene analogues’ have a 2‐benzylbenzimidazole core and belong to the group of new synthetic opioids (NSOs), which are among the fastest‐growing class of new psychoactive substances (NPS) worldwide [1]. Originally studied in the 1950s–60s by the Swiss pharmaceutical company Chemische Industrie Basel (CIBA) for their potential as analgesics, these compounds are structurally unrelated to traditional opiates or fentanyl [2]. However, they mimic their effects. Hence, nitazenes also bind to the classical opioid receptors: μ‐ (MOR), δ‐ (DOR), κ‐ (KOR) and nociception opioid receptor. In a forensic context, MOR is the main receptor of interest, since it regulates effects such as antinociception, constipation, euphoria and respiratory depression, the last one being the main cause of fatal NSO overdoses [3].

On 1 July 2025 the Ministry of Public Security of the People's Republic of China, the National Health Commission of the People's Republic of China and the National Medical Products Administration placed nitazene analogues under national control using a generic definition [4, 5]. This ban is expected to have far‐reaching effects on the NPS market. It may be comparable to those observed after the class‐wide ban of synthetic cannabinoid receptor agonists in China in 2021 [6], resulting in the emergence of semi‐synthetic cannabinoids in 2022 [7].

**FIGURE 1:**
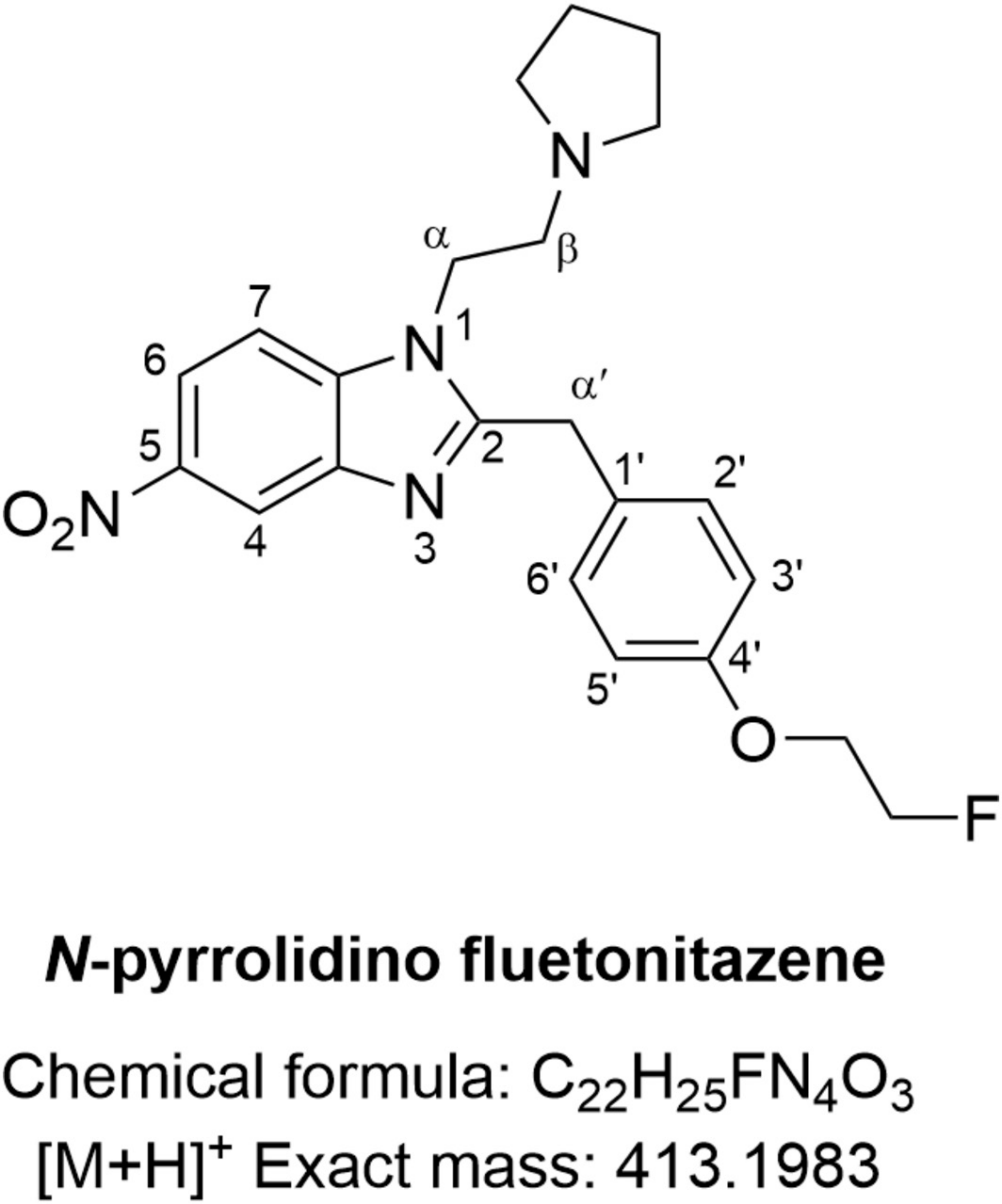
Chemical structure, chemical formula and monoisotopic mass of *N*‐pyrrolidino fluetonitazene.

*N*‐pyrrolidino fluetonitazene (*N*‐pyrrolidino‐4′‐(2‐fluoroethoxy) nitazene), also known as fluetonitazepyne is the fluorinated analogue of etonitazepyne (see Figure 1). It was first detected in the European Union in 2024 and in Switzerland in 2025 in postmortem samples. Recently, Kriikku et al. [8] reported on the quantification of *N*‐pyrrolidino fluetonitazene in postmortem blood and urine samples and found the metabolite 4′‐OH‐nitazepyne to be a suitable marker for general nitazene intoxication. However, they did not investigate the full biotransformation pathway of this compound.

The in vitro model pooled human liver microsomes (pHLM) is a widely applied model for investigating the phase I metabolism of NPS, particularly when no or only a few authentic human case samples are available [9, 10, 11, 12]. pHLM contain the major drug‐metabolising enzymes of the liver, especially cytochrome P450 isoforms [13]. To account for interindividual variability in enzyme expression, microsomes from multiple donors (up to 200 individuals) are pooled in pHLM‐based metabolism studies. Compared to in vivo studies, this model avoids ethical constraints and, in contrast to animal models, it provides human‐specific metabolic information without interspecies differences [14]. Other advantages include low cost, simple handling, easy storage, long‐term stability of frozen enzyme activities and that it is a well characterized and established in vitro model.

The aim of the present study is the investigation of the in vivo and in vitro metabolism of *N*‐pyrrolidino fluetonitazene. For the in vivo metabolism authentic postmortem blood and urine samples are analysed using liquid chromatography‐high resolution tandem mass spectrometry (LC‐HR‐MS/MS). The in vitro phase I metabolites are generated in pHLM. Based on these results a metabolic pathway of *N*‐pyrrolidino fluetonitazene and possible biomarkers for forensic casework are proposed. Furthermore, the quantification of the case samples is performed using liquid chromatography tandem mass spectrometry (LC–MS/MS) and the MOR affinity of *N*‐pyrrolidino fluetonitazene is investigated using a novel LC–MS/MS competitive receptor affinity assay [15, 16].

## 2 Materials and Methods

### 2.1 Materials

The certified reference standard *N*‐pyrrolidino fluetonitazene was purchased from Cayman Chemicals (Chemie Brunschwig, Basel Switzerland), the certified reference standards fentanyl‐D_5_, ecgonine methyl ester‐D_3_ (EME‐D_3_), tramadol‐^13^CD_3_ and THC‐D_3_ from Cerilliant (Sigma‐Aldrich, Buchs, Switzerland) and the certified reference standard 3,4‐methylenedioxyethylamphetamine‐D_5_ (MDEA‐D_5_) from Lipomed (Arlesheim, Switzerland). Methanol (absolute, HPLC grade) was obtained from Biosolve (Chemie Brunschwig, Basel, Switzerland) and acetonitrile (HPLC gradient grade, 99.9%) from Acros Organics (Chemie Brunschwig, Basel, Switzerland). Ethylene glycol (puriss, p.a., ≥ 99.5%), formic acid (puriss. p. a. for HPLC, 50% in water, 49%–51%), ammonium sulfate and ammonium formate were obtained from Honeywell Fluka (Sigma‐Aldrich, Buchs, Switzerland). Dichloromethane and ethyl acetate were purchased from Supelco Merck (Grogg, Stettlen‐Deisswil, Switzerland). 2‐propanol was purchased from Fisher Analytic (Chemie Brunschwig, Basel, Switzerland). Superoxide dismutase (7002 units/mg protein), sodium hydroxide, magnesium chloride and ammonium formate were obtained from Sigma Aldrich (Buchs, Switzerland). BGTurbo β‐glucuronidase from KURA Biotech (Atlanta, Georgia) was purchased from Specialty Diagnostix (Passau, Germany). Ultrapure water was produced in‐house with a Direct‐Q water purification system from Millipore (Zug, Switzerland). pHLM (150 donors, 20 mg/mL) were obtained from Corning (Wiesbaden, Germany) and the NADPH‐regenerating solutions A/B were purchased from Promega (Dübendorf, Switzerland). Blank urine was donated by the last author and blank blood was purchased from the blood bank (Interregionale Blutspende SRK, Bern, Switzerland). Both were tested with the routine screening LC‐HR‐MS/MS method to ensure the absence of any medicinal or recreational drugs. Human MOR (hMOR) membrane preparations in CHO‐K1 cells were purchased from Revvity (Zurich, Switzerland). The synthetic opioid peptide [D‐Ala^2^, *N*‐MePhe^4^, Gly^5^‐ol]‐enkephalin acetate salt (DAMGO) was purchased from VWR (Dietikon, Switzerland).

Authentic samples (femoral and cardiac blood and urine) came from an autopsy case with suspected consumption of a nitazene derivative acquired via the internet.

### 2.2 In Silico Metabolite Predictions

In silico metabolite predictions to assist the evaluation of the LC‐HR‐MS/MS data were performed using GLORYx, an open‐access software collaboratively developed by the University of Vienna (Austria) and the University of Hamburg (Germany) [17, 18]. Based on the Simplified Molecular Input Line Entry System (SMILES), GLORYx predicts phase I and phase II human metabolites and assigns each metabolite a prediction score (between 0 and 1), reflecting its likelihood of formation. Subsequent metabolites were predicted by using the SMILES of first predicted metabolites [11]. The predicted metabolites were added to a list for data evaluation.

### 2.3 In Vitro Metabolism Assay for pHLM Incubations

The microsomal in vitro experiments to generate phase I metabolites were performed as previously published [9, 19]. In brief (indicated concentrations are final concentrations): potassium phosphate buffer (100 mM), deionized water, magnesium chloride (5 mM), superoxide dismutase (200 units/mL), NADPH regenerating solution A (NADP^+^ and glucose‐6‐phosphate) and B (glucose‐6‐phosphate dehydrogenase), pHLM (1 mg/mL) and *N*‐pyrrolidino fluetonitazene (15 μM) were added into a reaction tube. Negative controls without pHLM and blanks without *N*‐pyrrolidino fluetonitazene were processed accordingly. Incubations were performed at 37°C and stopped after 1, 2, 4 and 6 h, respectively, by the addition of ice‐cold acetonitrile. An ISTD mix (5.0 μg/mL THC‐D_3_, 0.5 μg/mL EME‐D_3_ and 0.5 μg/mL tramadol‐^13^CD_3_) was added to the samples, followed by shaking and centrifugation at 17000 g for 10 min at 8°C. The supernatant was transferred to an autosampler vial and evaporated at 50°C under nitrogen. Samples were either stored at −20°C until analysis or directly reconstituted in deionized water/acetonitrile with 0.1% formic acid and 1 M ammonium formate (97.5:2.5, v/v).

### 2.4 Investigation of Authentic Samples

Blood and urine were sampled from an autopsy case. A man, 41 years old, was found in his apartment maximum 2 to 3 days after his death. At the scene, police seized a small bottle labelled “synthetic opioid” containing a few millilitres of a slightly yellow liquid. Since the deceased had a history of drug offences, authorities suspected a toxicological cause.

### 2.5 Qualitative Analysis of the Seized Liquid

GC–MS and LC‐HR‐MS/MS analysis of the diluted liquid was carried out to identify the suspected synthetic opioid. Preliminary identification in the nonderivatized sample by GC–MS suggested the presence of *N*‐pyrrolidino fluetonitazene based on the dataset 24/ADB‐106 provided by the Adebar Network. LC‐HR‐MS/MS (Orbitrap) suggested a compound with a protonated molecule with *m/z* 413.1975.

For confirmation, the analytical reference standard (from Cayman Chemicals) was analysed, and retention times and mass spectra in the GC–MS and the LC‐HR‐MS/MS revealed identical results for the reference and the unknown compound.

### 2.6 Preparation of Authentic Blood Sample for Metabolism Analysis and Quantification

Blood samples were prepared in duplicate for both the biotransformation investigation and for quantification using protein precipitation. A volume of 500 μL were extracted for the biotransformation experiments, and 190 μL were extracted for quantitative analysis. In short, ISTD solution (biotransformation: 5 μg/mL THC‐D_3_, 0.5 μg/mL EME‐D_3_, 0.5 μg/mL Tramadol‐^13^CD_3_; quantification: fentanyl‐D_5_) was added to the samples. Quantification was performed using a standard‐addition method to compensate for matrix effects: one sample without spiking and two samples spiked with two different concentration levels (0.5 ng/mL, 1 ng/mL in methanol) of *N*‐pyrrolidino fluetonitazene standard, respectively, were extracted. For protein precipitation acetonitrile was added in a ratio of 3:1 (v/v) and the samples were shaken for 10 min. This was followed by centrifugation at 17000 g at 8°C for 10 min. The supernatant was transferred into an autosampler vial and evaporated to dryness under nitrogen at 50°C. Samples were reconstituted in 100 μL deionized water/acetonitrile with 0.1% formic acid (97.5:2.5, v/v).

### 2.7 Preparation of Authentic Urine Sample for the Identification of Metabolites

For the identification of biotransformation products in urine, duplicate samples were prepared as follows: 10 μL of ISTD solution (5 μg/mL THC‐D_3_, 0.5 μg/mL EME‐D_3_ and 0.5 μg/mL tramadol‐^13^CD_3_) was added to duplicates of 1 mL of authentic urine sample and blank urine samples, respectively. Then, 1 mL of phosphate buffer pH 6 and 10 μL of β‐glucuronidase were added to the samples, followed by incubation at 37°C for 2 h. 2 mL of 30% ammonium sulfate solution was added to the samples and the pH was adjusted to 8–9 via addition of roughly 200 μL 1 M NaOH solution [20]. The samples were extracted twice using an equal volume of organic phase (dichloromethane: *2*‐propanol: ethyl acetate, 1:1:3, v/v/v), followed by vigorous shaking for 10 min and centrifugation at 3800 g at 10°C for 10 min to achieve phase separation. The organic phase was evaporated under nitrogen at 50°C, dissolved in 1 mL of organic extraction mix and transferred to autosampler vials. The samples were evaporated once more and reconstituted in 100 μL mobile phase (water/acetonitrile with 0.1% formic acid, 97.5:2.5, v/v) for analysis.

### 2.8 Identification and Analysis of Metabolites by LC‐HR‐MS/MS

For the investigation of the metabolism, a Shimadzu Nexera HPLC system (Shimadzu, Muttenz, Switzerland) coupled to a Sciex TripleTOF 6600 + system with Analyst software TF 1.8.1 (Sciex, Toronto, Canada) and LabSolution 5.110 (Shimadzu, Muttenz, Switzerland) was used. MS data were acquired in positive ionization mode with curtain gas and ion source gases 1 and 2 all set to 55 arbitrary units, an ion spray voltage of 5000 V and a source temperature of 650°C. Data were collected in information dependent data acquisition (IDA) and SWATH mode (‘SWATH_1’: 26 windows in a survey scan from 50 to 950 *m/z* with a width of 35 Da; ‘SWATH_2’: 9 windows around the *m/z* of expected metabolites with a width of 1 Da).

For both the authentic case samples and the pHLM samples, the following chromatographic conditions were applied: chromatographic separation was performed at a flow rate of 0.35 mL/min on a reversed‐phase Kinetex C8 column, 2.6 μm, 100 Å, 100 × 2.1 mm (Phenomenex, Aschaffenburg, Germany) at 40°C. The following gradient with eluent A (0.1% formic acid in water) and eluent B (0.1% formic acid in acetonitrile) was applied: 0–1 min: 2.5% B, 1–25 min: 2.5% –30% B, 25–26 min: 30%–97.5% B, 26–29 min: 97.5% B, 29–29.1 min: 97.5%–2.5% B and 29.1–35 min: 2.5% B. The injection volumes were 2 μL for pHLM samples and 10 μL for urine and blood samples. A calibration sample was measured at the beginning of the batch and after every fifth sample.

### 2.9 Quantification of *N*‐Pyrrolidino Fluetonitazene in Blood and Urine Samples

Quantification of *N*‐pyrrolidino fluetonitazene in blood and urine was performed with a Sciex 5500 + triple quadrupole mass spectrometer equipped with a Turbo V Ion Source coupled to a Shimadzu HPLC system. For separation, a Kinetex F5 column (2.6 μm, 100 × 2.1 mm, Phenomenex) at 50°C was used. Eluents were the same as mentioned above, and the following elution gradient with a flow rate of 0.25 mL/min was applied: 0–1 min 2.5% B, 1–5 min 2.5%–40% B, 5–7 min 40% B, 7–9 min 40%–97.5% B, 9–10.5 min 97.5% B, 10.5–10.6 min 97.5%–2.5% B and 10.6–12.5 min 97.5% B. The total runtime was 12.5 min and the injection volume was 5 μL. The mass spectrometer was operated in multiple reaction monitoring (MRM) mode in positive ionization mode, with the transitions listed in Table 1, optimized for *N*‐pyrrolidino fluetonitazene and the internal standard fentanyl‐D_5_. The autosampler temperature was set to 8°C and the curtain gas (nitrogen) to a pressure of 40 psi, while ion source gases 1 and 2 (N_2_) were set to 40 psi and 60 psi, respectively. The ion source temperature was set to 600°C, with an ion spray voltage of +3500 V.

**TABLE 1:** Optimized parameters for the MRM experiments.

Analyte	Q1^a^ [amu]^c^	Q3^b^ [amu]^c^	DP^d^ [V]	CE^e^ [V]	CXP^f^ [V]
*N*‐pyrrolidino fluetonitazene	413.0	152.9^g^	30	35	8
		296.1	28	50	15
		107	25	75	20
Fentanyl‐D_5_	342.2	105	60	53	3
DAMGO	514.1	133.9^g^	60	60	20
		219.2	30	30	15
		453.0	40	25	10
MDEA‐D_5_	213.1	163.1	40	16	4

^a^ Q1: m/z of the precursor ion.

^b^ Q3: m/z of the fragment ion.

^c^ amu: Atomic mass unit.

^d^ DP: Declustering potential.

^e^ ce: Collision energy.

^f^ CXP: Collision cell exit potential.

^g^ Ion transition used for quantification.

### 2.10 MOR Affinity Assay Using DAMGO and LC–MS/MS

The human MOR (hMOR) binding affinity of *N*‐pyrrolidino fluetonitazene was tested, as previously described [15], using *the competitive LC–MS/MS binding assay* with DAMGO. The assay incubation and measurements were conducted in 96‐deep‐well plates (Thermo Fisher Scientific Cat. No 60180‐P133, Basel, Switzerland). All assay preparations were performed on ice. The assay buffer consisted of 50 mM ammonium formate (pH 7.4 at 27°C). Dilutions for the test compound and reference compound (fentanyl), as well as the solution of DAMGO were prepared freshly in assay buffer on the day of the experiment. The test compound and reference compound (final concentration range: 31.6 to 0.00316 nM), DAMGO (2 nM) and the hMOR (5 μg protein/well) were incubated at 27°C for 60 min. The final assay volume was 200 μL. Reactions were stopped by transferring the whole assay volume to a MultiScreen filter plate (1.2 μM, Merck, Germany), which had been pre‐incubated with 0.1% PEI and washed twice with ice‐cold assay buffer using a vacuum manifold (MutliScreen 96‐well‐vacuum manifold, Merck, Germany). Samples were washed three times with ice‐cold assay buffer. Before elution with methanol, the ISTD MDEA‐D_5_ (0.25 ng/mL) was added. Samples were shaken for 10 min at room temperature and then transferred to HPLC vials containing 5 μL of ethylene glycol. Samples were then evaporated at 50°C under nitrogen followed by reconstitution in 200 μL mobile phase (water/acetonitrile, 97.5:2.5, v/v with 0.1% formic acid). Quantification of DAMGO was performed as previously described [16], using a10‐point calibration curve (0.024–1.798 ng/mL equivalent to 46.9 pM–3.5 nM) applying a linear 1/x^2^ weighted regression model. The same chromatographic method was applied as described in Section 2.9. The optimized parameters for the MRM experiments are listed in Table 1. The assay was performed on three different days, and each concentration was tested at least in duplicate per plate. At least one replicate of fentanyl (seven different concentrations, ranging from 1000 to 0.0316 nM) was measured during each experiment as a quality control.

### 2.11 Data Analysis

Raw data were processed using Analyst TF version 1.8.1 and PeakView embedded in Sciex OS 2.0.0.45330 (Sciex, Toronto, Canada). Analysis of the MOR receptor affinity assay data was performed with Microsoft Excel 2016 and GraphPad Prism version 10.4.1 (Boston, MA, USA). IC_50_ values were determined at the turning point of the sigmoidal graph (semilogarithmic scale of the horizontal axis) by using one‐site‐fit K_i_ binding model with nonlinear regression. Calculations were performed using a K_D_ value of 0.27 nM [21]. The corresponding K_i_ values were calculated using the Cheng‐Prusoff equation [22].

## 3 Results and Discussion

### 3.1 Identification of In Vitro Metabolites

The in vitro phase I metabolism of *N*‐pyrrolidino fluetonitazene was investigated via incubation of the parent compound with pHLM and compared against controls (no pHLM) and blanks (no drug). The generated metabolites were identified using LC‐HR‐MS/MS. Compounds with a peak height of fewer than 1000 counts per second in the extracted ion chromatogram were not included (see Figure S1). In HR‐MS/MS‐based metabolism workflows, tentative identification of novel metabolites is achieved by deriving molecular formulas from accurate mass measurements in combination with isotope pattern information [23]. This constrains the number of plausible candidates to a limited set. The obtained molecular formula can subsequently be used to retrieve candidate structures, for instance via lists of possible metabolite candidates potentially derived from in silico prediction [24]. Finally, candidate metabolites are further characterized based on characteristic MS/MS fragmentation patterns.

In total, eight metabolites of the parent compound were identified in the 1 h pHLM assay (denoted as **M1**–**M8**, arranged in order of decreasing signal intensity). It should be noted that, due to potential differences in ionization efficiency and matrix effects, the observed signal intensities may not accurately reflect the true relative abundance of the metabolites. An overview of the metabolites detected in vitro and their most abundant fragmentation ions in MS/MS experiments is provided in Table 2. Characteristic fragment ions containing the fluoroethyl moiety are indicated with an asterisk. A detailed list of all identified fragment ions for each metabolite detected in vitro is given in Table S1.

**TABLE 2:** Retention times, chemical formulas, mass errors, peak height intensities and most abundant fragment ions of *N*‐pyrrolidino fluetonitazene and its identified in vitro *and* in vivo metabolites after 1 h incubation with pHLM and in postmortem blood and urine samples, respectively. All values are averaged over triplicate experiments. For each fragment their relative percentage of the base peak is given as an index number. Ions labelled with an asterisk (*) represent relatively abundant fragments containing the characteristic fluoroethyl moiety. Compounds with peak heights of fewer than 1000 counts per second in the extracted ion chromatogram were not included, except for metabolite **M8**, since it was also detected in vivo.

Compound name	Biotransformation	Matrix	RT [min]	Chemical formula [M + H]^+^	Theoretical [M + H]^+^ [Da]	Measured [M + H]^+^ [Da]	Error [ppm]	Intensity [cps]	Fragment ions [*m/z*]
**N‐**pyrrolidino fluetonitazene	Parent drug	pHLM	16.61	C_22_H_26_FN_4_O_3_^+^	413.19835	413.1996	3.1	1.3 × 10^5^	98.0968 _***100***_
		Urine							153.0709 _***5***_ *
									107.0490 _***4***_
									56.0494 _***4***_
**M1**	Dehydrogenation	pHLM	15.61	C_22_H_24_FN_4_O_3_^+^	411.18270	411.1835	2.0	1.8 × 10^4^	96.0806 _***100***_
	(−2H)								153.0709 _***45***_ *
									107.0488 _***35***_
									296.1326 _***17***_ *
**M2**	*O*‐dealkylation	pHLM	10.04	C_20_H_23_N_4_O_3_^+^	367.17647	367.1766	0.3	5.5 × 10^3^	98.0962 _***100***_
	(−C_2_H_3_F)	Urine							107.0487 _***12***_
									56.0492 _***7***_
									250.1106 _***2***_
**M3**	*O*‐dealkylation &	pHLM	7.38	C_20_H_21_N_4_O_3_^+^	365.16082	365.1618	2.7	6.5 × 10^3^	96.0805 _***100***_
	Oxidation								107.0488 _***54***_
	(−C_2_H_3_F, −2H)								250.1105 _***31***_
									222.0791 _***16***_
**M4**	Oxidation	pHLM	23.96	C_22_H_24_FN_4_O_4_^+^	427.17761	427.1789 *	2.9	5.7 × 10^3^	112.0755 _***100***_
	(+O, −2H)								69.0335 _***4***_
									84.0804 _***2***_
**M5**	*N,N*‐bisdealkylation	pHLM	14.30	C_18_H_20_FN_4_O_3_^+^	359.1514	359.1520	1.8	1.9 × 10^3^	316.1098 _***100***_ *
	(−C_4_H_6_)								176.0454 _***44***_
									270.1163 _***33***_ *
									302.1071 _***21***_ *
**M6**	Hydrolytic deamination	pHLM	20.16	C_18_H_19_FN_3_O_4_^+^	360.13541	360.1360 *	1.6	1.2 × 10^3^	145.0757 _***77***_
	(−C_4_H_7_N, +O)								314.1430 _***67***_ *
									346.1324 _***42***_ *
									107.0485 _***26***_
**M7**	*O*‐dealkylation &	pHLM	11.42	C_16_H_16_N_3_O_4_^+^	314.11353	314.1145	3.0	1.1 × 10^3^	268.1211 _***100***_
	Hydrolytic deamination	Urine							145.0759 _***93***_
	(−C_6_H_10_FN, +O)								224.0943 _***58***_
									300.1108 _***55***_
**M8**	Oxidative ring opening and carboxylation	pHLM	15.91	C_22_H_26_FN_4_O_5_^+^	445.18817	445.1886	1	7.5 × 10^2^	130.0858 _***100***_
		Urine							112.0753 _***42***_
	(+2O)								87.0437 _***23***_
									316.1092 _***17***_ *
**M9**	*N*‐acetylation	Urine	7.73	C_24_H_30_FN_4_O_2_^+^	425.23473	425.2342*	−1.3	1.9 × 10^4^	98.0961 _***100***_
	(−O, +C_2_H_4_)	Femoral blood							153.0704 _***6***_ *
		Heart blood							107.0480 _***3***_
									56.0494 _***3***_

#### 3.1.1 Parent Compound—*N*‐Pyrrolidino Fluetonitazene

**FIGURE 2:**
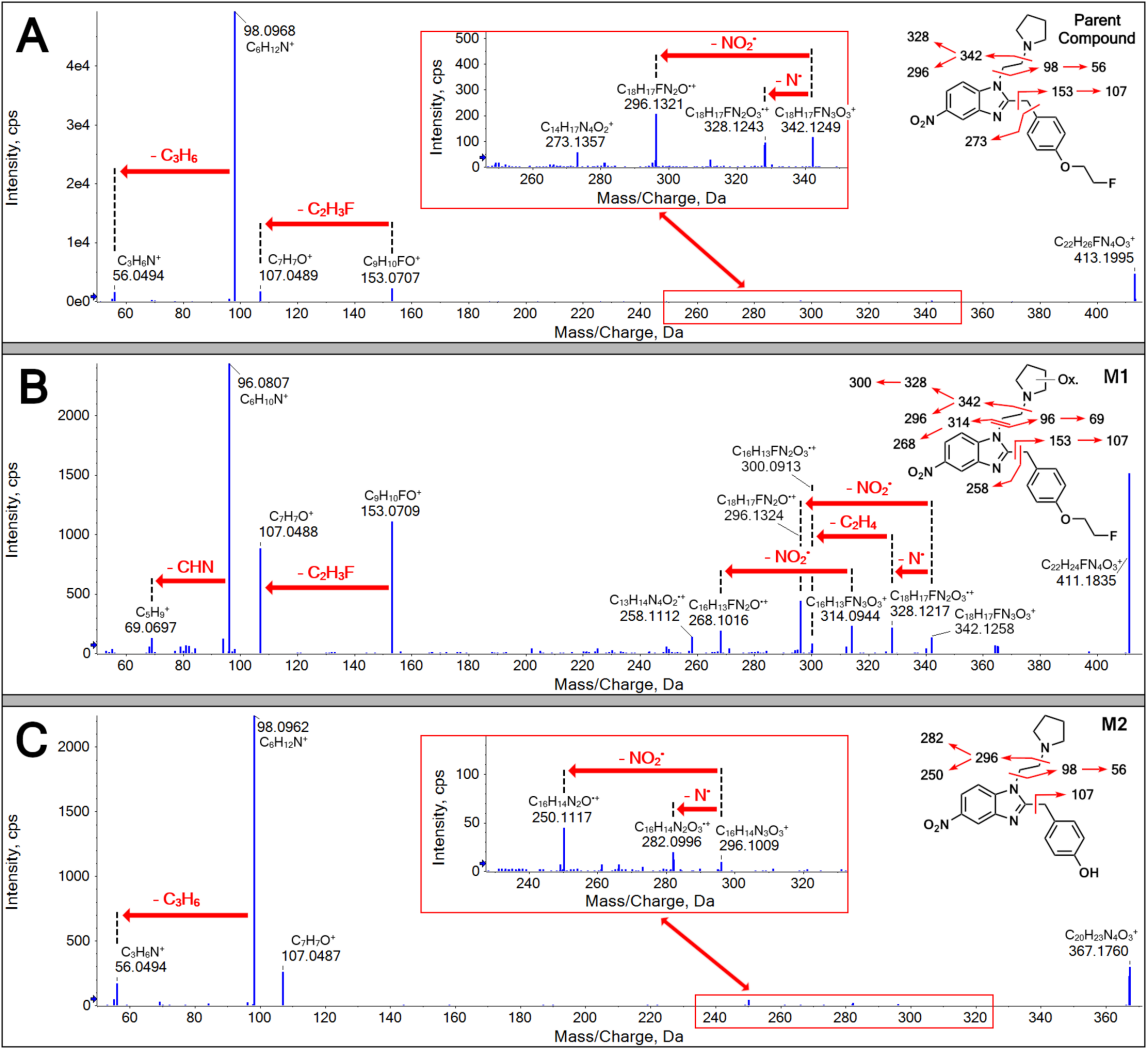
Product ion spectra of (A) *N*‐pyrrolidino fluetonitazene (parent compound) and its (B) dehydrogenated metabolite (**M1**) and (C) *O*‐dealkylated metabolite (**M2**) found in vitro by IDA experiments. Fragmentation of selected precursor ions was conducted with a CE of 35 eV and a CES of ±15 eV.

The parent compound *N*‐pyrrolidino fluetonitazene ([M + H]^+^ *m/z* 413.1995, C_22_H_26_FN_4_O_3_^+^, 2.8 ppm) eluted at 16.61 min. The corresponding MS/MS spectrum can be seen in Figure 2A with red arrows indicating fragmentation transitions between related fragment ions. The most intense fragment *m/z* 98.0968 (C_6_H_12_N^+^) resulted from α‐cleavage of the C–N bond on the nitrogen‐containing side chain, and it is a characteristic fragment found in the *N*‐pyrrolidino class of nitazenes [25]. Further fragmentation of the pyrrolidine ring through neutral loss of C_3_H_6_ yielded the fragment *m/z* 56.0494 (C_3_H_6_N^+^). α'‐cleavage of the C–C bond on the alkoxy benzyl side chain resulted in fragment *m/z* 153.0707 (C_9_H_10_FO^+^), while further loss of the fluoroethyl substituent generated fragment *m/z* 107.0489 (C_7_H_7_O^+^). The latter is encountered in many mass spectra of various nitazene analogues [25, 26, 27, 28, 29]; however, whether its chemical structure resembles a substituted cyclohexadiene or a hydroxylated tropylium ion is still debated in literature [29, 30, 31]. The fragment *m/z* 153.0707 (C_9_H_10_FO^+^) correlates with the substitution on the alkoxy benzyl side chain and thus confirms the suspected fluoroethyl moiety. It is therefore a characteristic fragment for *N*‐pyrrolidino fluetonitazene. α‐cleavage was the dominant fragmentation pathway, as demonstrated by the intensity of the fragment *m/z* 98.0968. However, γ‐cleavage of the C–N bond on the nitrogen‐containing side chain was also observed, resulting in a fragment of *m/z* 342.1249 (C_18_H_17_FN_3_O_3_^+^). Loss of either a nitrogen radical or a nitro radical yielded the fragments *m/z* 328.1243 (C_18_H_17_FN_2_O_3_^•+^) and *m/z* 296.1321 (C_18_H_17_FN_2_O^•+^), respectively. The comparatively low intensity of this fragmentation pathway suggests that the positive charge on the intact molecule is much more likely to be found on the pyrrolidine ring as opposed to the benzimidazole core structure. This is a recurring pattern in pyrrolidine‐containing nitazene analogues [25, 27, 28, 29]. The fragment *m/z* 273.1357 (C_14_H_17_N_4_O_2_^+^) was generated by β'‐cleavage of the C–C bond on the alkoxy benzyl side chain and still contains the pyrrolidine ring, but its low intensity suggests further fragmentation with loss of the fragment *m/z* 98.0968 (C_6_H_12_N^+^).

#### 3.1.2 Metabolite **M1**—Oxidation of the Pyrrolidine Ring

Metabolite **M1** ([M + H]^+^ *m/z* 411.1835, C_22_H_24_FN_4_O_3_^+^, 2 ppm) was generated by dehydrogenation, forming a double bond in the pyrrolidine ring of the parent compound and eluted at 15.61 min. Its MS/MS spectrum is shown in Figure 2B. Presence of the fragment *m/z* 96.0807 (C_6_H_10_N^+^) but the absence of the fragment *m/z* 98.0968 (C_6_H_12_N^+^) suggests that the dehydrogenation occurred on the pyrrolidine ring. This is supported by the fragment *m/z* 153.0709 (C_9_H_10_FO^+^) generated by the intact alkoxy benzyl side chain, indicating that the fluoroethyl substituent stayed intact. Furthermore, fragments of the benzimidazole core are consistent with those seen in the parent compound MS/MS spectrum, in particular fragments *m/z* 342.1259 (C_18_H_17_FN_3_O_3_^+^), *m/z* 328.1217 (C_18_H_17_FN_2_O_3_^•+^, here with its decomposition product *m/z* 300.0913 (C_16_H_13_FN_2_O_3_^•+^)) and *m/z* 296.1324 (C_18_H_17_FN_2_O^•+^), confirming that no biotransformation took place at this site. One interesting aspect that we observed in the MS/MS spectrum of **M1** is the absence of fragment *m/z* 56.0494 (C_3_H_6_N^+^), a decomposition product of the pyrrolidine ring. It appears that the specific position on the ring which was targeted by the biotransformation was crucial for this particular fragmentation pathway. As a result, the pathway was inhibited. Instead, fragment *m/z* 69.0697 (C_5_H_9_^+^) was observed with a negative mass shift of *m/z* 27.011 (CHN), an entirely different decomposition product.

#### 3.1.3 Metabolite **M2**—*O*‐dealkylation

Dealkylation on the alkoxy benzyl side chain of *N*‐pyrrolidino fluetonitazene yielded metabolite **M2** with a signal of *m/z* 367.1760 ([M + H]^+^ C_20_H_23_N_4_O_3_^+^, −1.3 ppm). The metabolite eluted at 10.04 min and its MS/MS spectrum can be seen in Figure 2C. The MS/MS spectrum shows many similarities to the MS/MS spectrum of the parent compound (Figure 2A). The presence of the characteristic two fragments *m/z* 98.0962 (C_6_H_12_N^+^) and *m/z* 56.0494 (C_3_H_6_N^+^) demonstrates that the pyrrolidine ring remained intact. A signal at *m/z* 107.0487 (C_7_H_7_O^+^) together with the absence of the fragment *m/z* 153.0707 (C_9_H_10_FO^+^) confirms that dealkylation occurred at the fluoroethyl side chain. Trace signals at *m/z* 296.1009 (C_16_H_14_N_3_O_3_^+^), *m/z* 282.0996 (C_16_H_14_N_2_O_3_^•+^) and *m/z* 250.1117 (C_16_H_14_N_2_O^•+^) provide further evidence, as they are consistent with fragments found in the parent compound MS/MS spectrum after loss of the fluoroethyl moiety with *m/z* 46.0219 (*m/z* 342.1249, *m/z* 328.1243 and *m/z* 296.1321 respectively).

#### 3.1.4 Metabolite **M3**—*O*‐dealkylation and Dehydrogenation at the Pyrrolidine Ring

**FIGURE 3:**
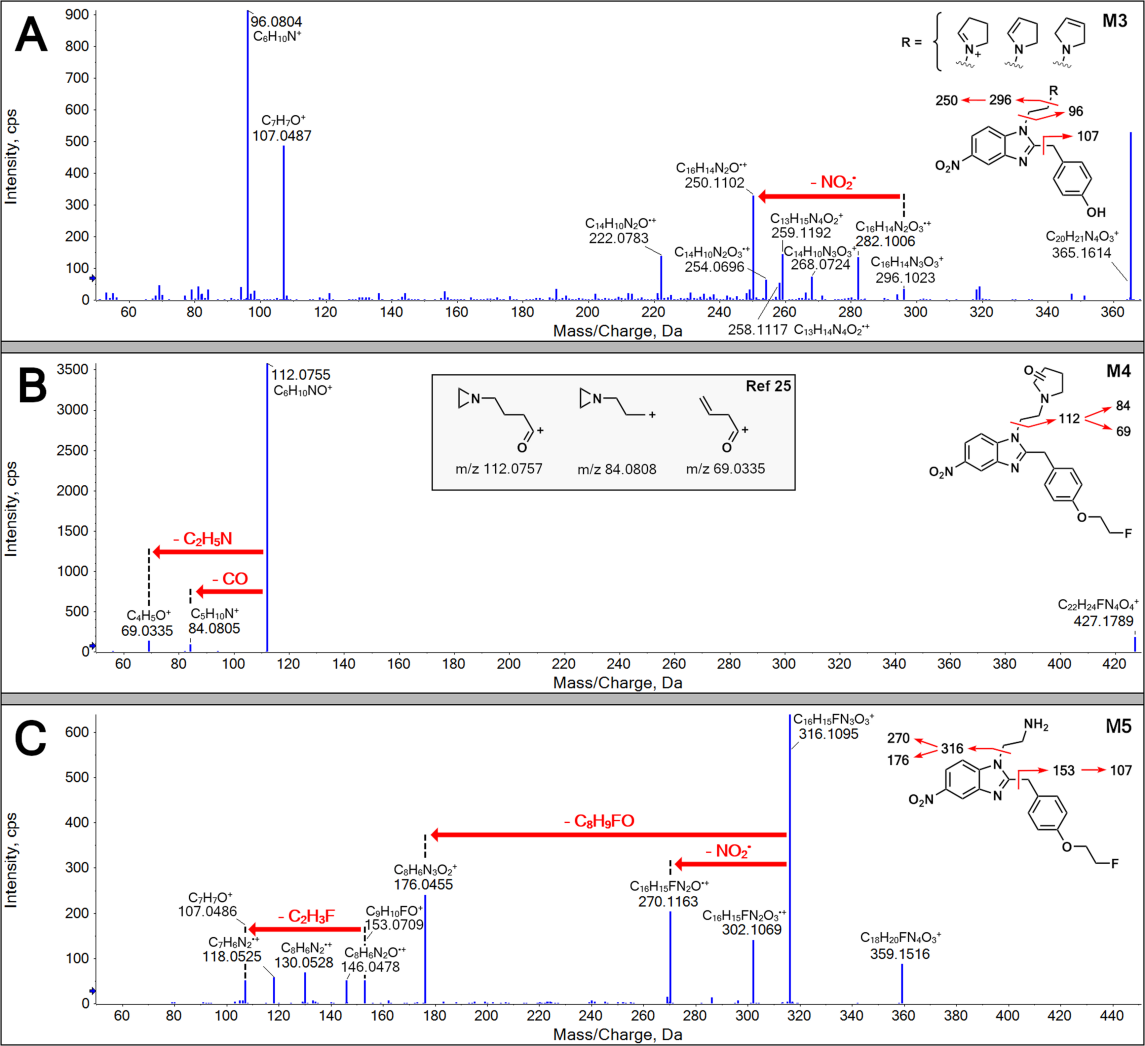
Product ion spectra of (A) *O*‐dealkylation and dehydrogenation metabolite (**M3**), (B) oxidation metabolite (**M4**) and (C) N,N‐bisdealkylation metabolite (**M5**) generated in vitro by IDA. Fragmentation of selected precursor ions was conducted with a CE of 35 eV and a ceS of ±15 eV. The fragment ion structures in (B) were taken from Magny et al. [25].

The combination of *O*‐dealkylation and dehydrogenation yielded metabolite **M3** ([M + H]^+^ *m/z* 365.1614, C_20_H_21_N_4_O_3_^+^, 1.6 ppm), which eluted at 7.38 min. The corresponding MS/MS spectrum can be seen in Figure 3A. Similar fragmentation patterns compared to metabolites **M1** and **M2** (dehydrogenation and *O*‐dealkylation, respectively, Figure 3B,C) can be observed. The presence of the fragment *m/z* 96.0804 (C_6_H_10_N^+^), instead of *m/z* 98.0968 (C_6_H_12_N^+^), absence of the fragment *m/z* 56.0494 (C_3_H_6_N^+^) and a general increase in the relative intensities of other fragments confirm that the dehydrogenation took place on the pyrrolidine ring. The fragment *m/z* 107.0487 (C_7_H_7_O^+^), which is generated by fragmentation of the fluoroethyl side chain, and a lack of fragment *m/z* 153.0707 (C_9_H_10_FO^+^) demonstrate that the oxygen on the 4′‐position of the aromatic ring was dealkylated. Fragments generated by γ‐cleavage of the C–N bond of the nitrogen‐containing side chain were consistent with fragments observed in the parent compound MS/MS spectrum (Figure 2A).

#### 3.1.5 Metabolite **M4**—Oxidation of the Pyrrolidine Ring

The formation of metabolite **M4** at *m/z* 427.1789 ([M + H]^+^ C_22_H_24_FN_4_O_4_^+^, 3.0 ppm) was attributed to oxidation at the pyrrolidine ring and it eluted at 23.96 min. The MS/MS spectrum of **M4** can be seen in Figure 3B. The absence of a fragment at *m/z* 98.0968 (C_6_H_12_N^+^) but the presence of a fragment at *m/z* 112.0755 (C_6_H_10_NO^+^), corresponding to α‐cleavage, demonstrate that the biotransformation occurred on the pyrrolidine ring. Compared to the mass spectra of previously discussed metabolites, fragmentation pathways other than those involving the pyrrolidine ring were virtually non‐existent. This loss of information prevented a definitive characterization of metabolite **M4**. However, recent publications that investigated the metabolism of structurally related nitazenes reported analog metabolites to **M4** [27, 28]. Two research groups found the proposed biotransformation (+O, −2H) for **M4**, for the compounds *N*‐pyrrolidino etonitazene [27] as well as *N*‐pyrrolidino metonitazene (“metonitazepyne”) and *N*‐pyrrolidino protonitazene (“protonitazepyne”) [28], respectively. In all three cases, these metabolites eluted after the parent drug in reversed‐phase chromatography, which was also the case for **M4**. It is currently unclear why this particular biotransformation increases interactions of the metabolite with the reverse phase column material.

**FIGURE 4:**
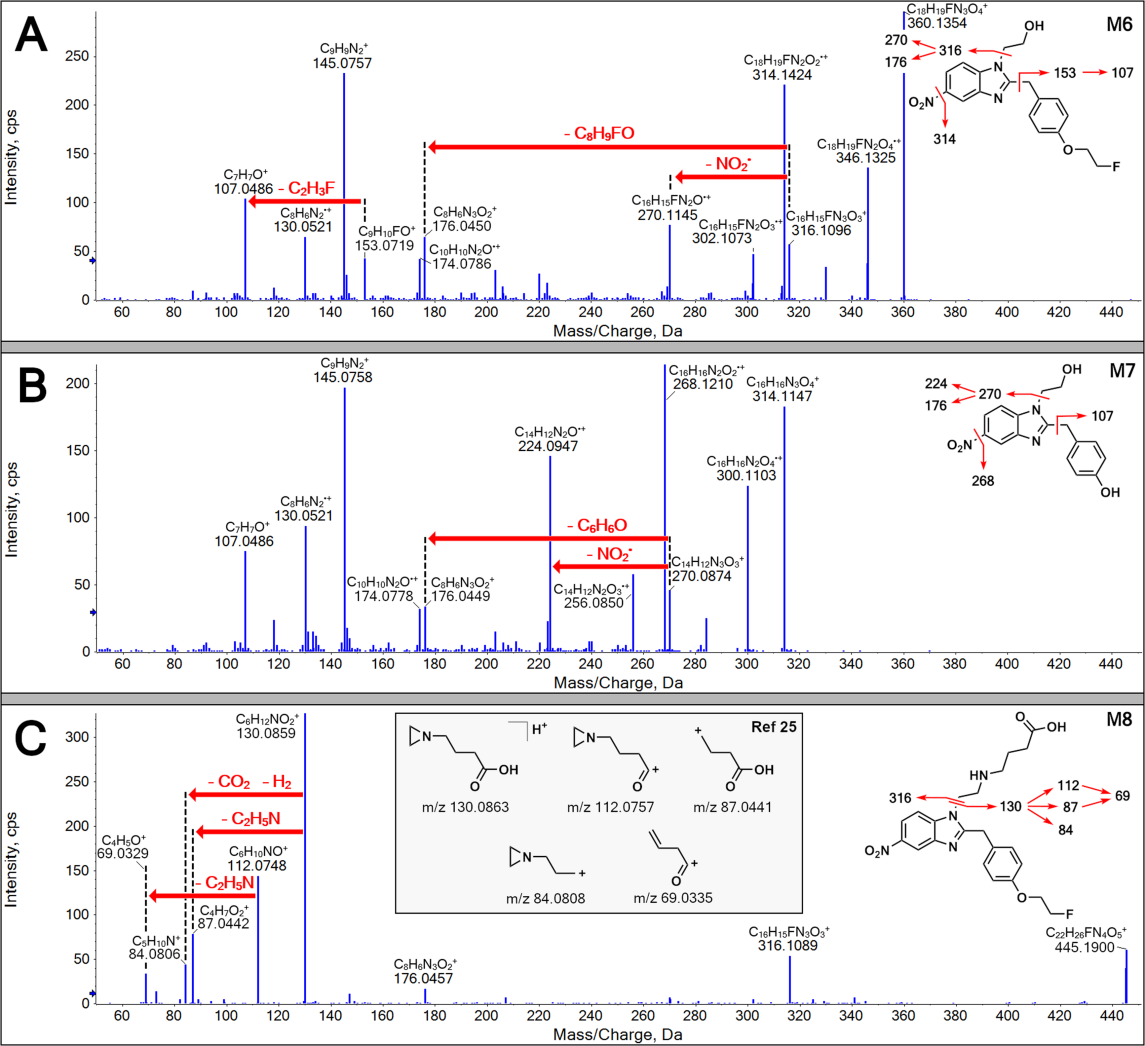
IDA Product ion spectra of in vitro metabolites of *N*‐pyrrolidino fluetonitazene as a result of (A) hydrolytic deamination (**M6**), (B) *O*‐dealkylation and hydrolytic deamination (**M7**) and (C) oxidative ring opening and carboxylation (**M8**). Fragmentation of selected precursor ions was conducted with a ce of 35 eV and a ceS of ±15 eV. The fragment ion structures in (C) were taken from Magny et al. [25].

The metabolites **M4** and **M8** show similar fragmentation patterns (see Figures 3B and 4C), with fragments of *m/z* 112.0755 (C_6_H_10_NO^+^), *m/z* 84.0805 (C_5_H_10_N^+^) and *m/z* 69.0335 (C_4_H_5_O^+^) occurring in the MS/MS spectra of both metabolites. Recently, Magny et al. [25] reported on the in vivo metabolism of *N*‐pyrrolidino protonitazene, a structural analogue of *N*‐pyrrolidino fluetonitazene and identified a *N*‐butanoic acid metabolite, which was also found in this study as metabolite **M8** (Figure 4C). Comparison of the mass MS/MS spectrum of **M4** with the *N*‐butanoic acid metabolite described by Magny et al. [25] demonstrated a high degree of spectral similarity. All three fragments observed in **M4** (*m/z* 112.0755 (C_6_H_10_NO^+^), *m/z* 84.0805 (C_5_H_10_N^+^) and *m/z* 69.0335 (C_4_H_5_O^+^)) were also observed in the *N*‐butanoic acid metabolite of *N*‐pyrrolidino protonitazene. Furthermore, fragments that included an intact carboxylic acid group, namely *m/z* 130.0863 (C_6_H_12_NO_2_^+^) and *m/z* 87.0441 (C_4_H_7_O_2_^+^), were not observed in **M4**, since only one oxygen was added to the parent drug. The authors also proposed structures for these fragments, which are illustrated in Figure 3B. The main fragment *m/z* 112.0756 (C_6_H_10_NO^+^) was suggested to be aziridine linked to a butyl chain with a terminal carbonyl group bearing a positive charge. The mass shifts between the fragments observed in this study corresponded to loss of CO (*m/z* 112.0755 to *m/z* 84.0805) and loss of aziridine (*m/z* 112.0755 to *m/z* 69.0335), respectively, both highly feasible fragmentation pathways based on the proposed structures. We therefore propose that **M4** is an intermediate of **M8**, which is an *N*‐butanoic acid metabolite. Similarly, Berardinelli et al. [11] reported in their investigation of the in vitro and in vivo metabolism of dipyanone, a synthetic opioid containing a pyrrolidine ring, α‐hydroxylation as a key step leading to ring opening via a hemiaminal intermediate. The exact position of oxidation cannot be determined. However, a similar reaction was observed in the metabolism of nicotine to cotinine. Gorrod and Aislaitner [32] proposed the formation of a γ‐lactam from a tertiary amine via an iminium ion intermediate. The resulting lactam is in tautomeric equilibrium with its lactim form where the nitrogen bears a positive charge, which might be a possible explanation for the intense signals generated by the pyrrolidine ring and suppression of other fragmentation pathways.

#### 3.1.6 Metabolite **M5**—*N,N*‐bisdealkylation

Metabolite **M5** ([M + H]^+^ *m/z* 359.1516, C_18_H_20_FN_4_O_3_^+^, 0.6 ppm) was identified as the product of *N,N*‐bisdealkylation of the parent drug. The compound eluted at 14.30 min and the corresponding MS/MS spectrum can be seen in Figure 3C. The most intense signal at *m/z* 316.1095 (C_16_H_15_FN_3_O_3_^+^) was generated by α‐cleavage of the C–N bond on the pyrrolidine‐containing side chain and is a fragmentation step typically observed in other nitazene analogues [10, 27]. The potential signal of the cleaved ethylamine moiety was not observed since the MS/MS spectrum window was restricted to signals ≥ 50 Da. Loss of a nitrogen radical, a nitro radical or the alkoxy benzyl side chain from this fragment yielded signals at *m/z* 302.1069 (C_16_H_15_FN_2_O_3_^•+^), *m/z* 270.1163 (C_16_H_15_FN_2_O^•+^) and *m/z* 176.0455 (C_8_H_6_N_3_O_2_^+^), respectively. Fragment *m/z* 270.1163 (C_16_H_15_FN_2_O^•+^) potentially undergoes further decomposition to produce fragments *m/z* 118.0525 (C_7_H_6_N_2_^•+^) upon loss of the alkoxy benzyl side chain and *m/z* 146.0478 (C_8_H_6_N_2_O^•+^) after loss of fluoroethyl and C_6_H_6_. Loss of a nitro radical from fragment *m/z* 176.0455 (C_8_H_6_N_3_O_2_^+^) yielded a signal at *m/z* 130.0528 (C_8_H_6_N_2_^•+^). Finally, the familiar α'‐cleavage of the C–C bond on the alkoxy benzyl side chain generated fragments *m/z* 153.0709 (C_9_H_10_FO^+^) and *m/z* 107.0486 (C_7_H_7_O^+^) after further loss of fluoroethyl.

Removing the pyrrolidine ring from the parent drug significantly altered the fragmentation patterns in the MS/MS spectrum. The majority of signals were generated by the benzimidazole core in various states of decomposition, with a preferred first step of removing the ethylene amine moiety. In comparison, the intensities of fragments generated via the α'‐pathway were surprisingly low, suggesting that the positive charge was predominantly carried by the benzimidazole core.

#### 3.1.7 Metabolite M6—Hydrolytic Deamination of the Pyrrolidine Ring

The signal of metabolite **M6** at *m/z* 360.1354 ([M + H]^+^ C_18_H_19_FN_3_O_4_^+^, 1.6 ppm) was identified as hydrolytic deamination of the parent drug. The compound eluted at 20.16 min. The MS/MS spectrum of **M6** (Figure 4A) has many similarities to the MS/MS spectrum of **M5** (*N,N*‐bisdealkylation, Figure 3C). Absence of a signal at *m/z* 98.0968 (C_6_H_12_N^+^) indicated loss of the pyrrolidine ring. Previously observed fragment *m/z* 316.1096 (C_16_H_15_FN_3_O_3_^+^) (compare **M5**, Figure 3B), once again further decomposed to *m/z* 302.1073 (C_16_H_15_FN_2_O_3_^•+^, loss of N^•^), *m/z* 270.1145 (C_16_H_15_FN_2_O^•+^, loss of NO_2_^•^) and *m/z* 176.0450 (C_8_H_6_N_3_O_2_^+^, loss of the alkoxy benzyl side chain), which could undergo further fragmentation by loss of a nitrogen radical to *m/z* 130.0521 (C_8_H_6_N_2_^•+^). Previously observed α'‐cleavage of the C–C bond on the alkoxy benzyl side chain yielded *m/z* 153.0719 (C_9_H_10_FO^+^) and *m/z* 107.0486 (C_7_H_7_O^+^) upon loss of a fluoroethyl moiety.

#### 3.1.8 Metabolite **M7**—*O*‐dealkylation and Hydrolytic Deamination of the Pyrrolidine Ring

Metabolite **M7** eluted at 11.42 min and resulted from *O*‐dealkylation and hydrolytic deamination ([M + H]^+^ *m/z* 314.1147, C_16_H_16_N_3_O_4_^+^, 3.7 ppm). The corresponding MS/MS spectrum is shown in Figure 4B. Owing to further biotransformation pathways, namely *O*‐dealkylation of metabolite **M6** (*m/z* 360.1354, C_18_H_19_FN_3_O_4_^+^, 1.6 ppm), a precursor ion mass that is 46.0213 Da lower occurred for **M7**. Consequently, the MS/MS spectrum of **M7** showed pronounced similarities to the MS/MS spectrum of M6 (Figure 4A). Moreover, previously identified fragments of **M6,** for which the fluoroethyl moiety was already cleaved, were also observed for **M7**. Furthermore, the absence of a signal at *m/z* 153.0707 (C_9_H_10_FO^+^) but presence of fragment *m/z* 107.0486 (C_7_H_7_O^+^) indicates that the alkoxy benzyl side chain was dealkylated. Hence, metabolite **M7** was generated by the combination of *O*‐dealkylation and hydrolytic deamination of the parent drug.

#### 3.1.9 Metabolite **M8**—Oxidative Ring Opening and Carboxylation

Metabolite **M8** was identified as the *N*‐butanoic acid metabolite ([M + H]^+^ *m/z* 445.1900, C_22_H_26_FN_4_O_5_^+^, 4.1 ppm) of the parent compound following ring opening and carboxylation of the pyrrolidine ring and eluted at 15.91 min. The MS/MS spectrum of **M8** can be seen in Figure 4C. Even though the peak height of the metabolite was below the cut‐off of 1000 counts per second after 1 h of incubation, it was nevertheless included since this metabolite was detected in vivo in the urine sample.

The most intense fragments were identified as decomposition products of the *N*‐butanoic acid chain. Some of these fragments were already seen in the MS/MS spectrum of metabolite **M4** (Figure 3B), indicating a certain structural resemblance between **M4** and **M8**. As mentioned earlier, Magny et al. [25], identified an *N*‐butanoic acid metabolite which aligned with parts of the observed fragmentation pattern of **M8** considering the mass shift of *m/z* 3.9749 (−CH_3_, +F) to account for the fluoroethyl substituent instead of the propyl group. Fragment *m/z* 130.0859 (C_6_H_12_NO_2_^+^) was proposed as aziridine connected to a butyl chain with a terminal carboxylic acid group and was generated by α‐cleavage of the C–N bond on the nitrogen‐containing side chain. Fragment *m/z* 87.0442 (C_4_H_7_O_2_^+^) was found with a negative mass shift of *m/z* 43.0417, which was attributed to removal of aziridine (−C_2_H_5_N). Loss of CO_2_ instead yielded the signal at *m/z* 84.0806 (C_5_H_10_N^+^), which was also observed in the MS/MS spectrum of metabolite **M4** after loss of CO respectively. Both fragmentations support the proposed structure, which features a terminal carboxylic acid and an aziridine. Loss of water from *m/z* 130.0859 (C_6_H_12_NO_2_^+^) generated fragment *m/z* 112.0748 (C_6_H_10_NO^+^), which was also observed in **M4**. Fragment *m/z* 69.0329 (C_4_H_5_O^+^) was identified as an advanced decomposition product of the *N*‐butanoic acid chain and was accessible via different pathways.

Additionally, two other fragments were observed for **M8**, fragment *m/z* 316.1089 (C_16_H_15_FN_3_O_3_^+^), which generated fragment *m/z* 176.0457 (C_8_H_6_N_3_O_2_^+^) upon loss of the alkoxy benzyl side chain. This represents a fundamental difference between the spectra of the two metabolites **M4** and **M8**, since only three fragments were observed for **M4**. This observation further strengthens the hypothesis that metabolite **M4** did not undergo ring opening, whereas metabolite **M8** did.

### 3.2 Evaluation of the In Vitro Metabolic Profile

**FIGURE 5:**
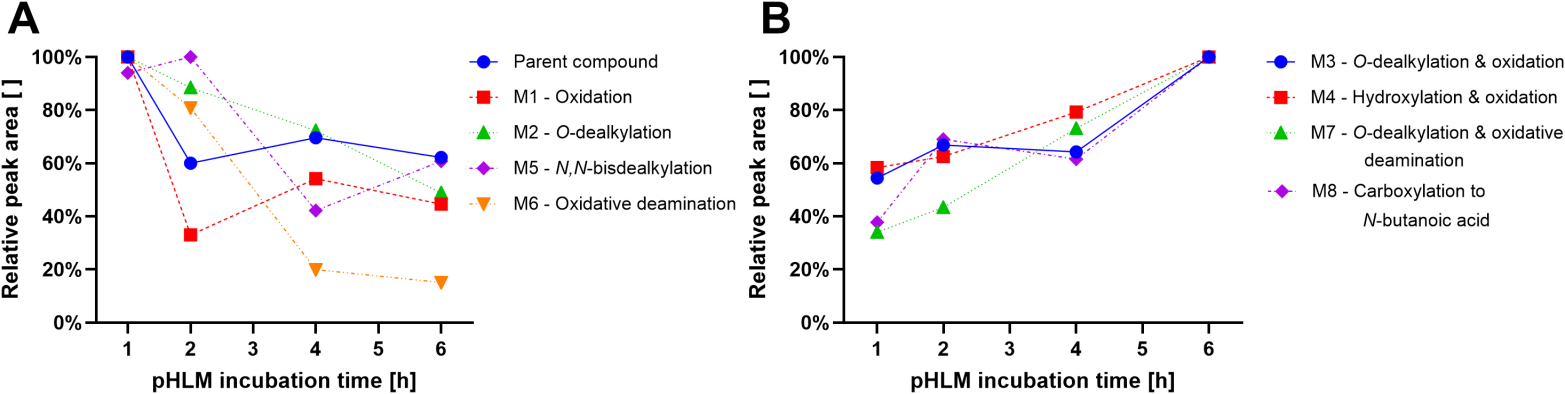
Relative peak areas of (A) intermediate and (B) final in vitro metabolites of N‐pyrrolidino fluetonitazene after 1, 2, 4 and 6 h incubation with pooled human liver microsomes (pHLM). All values are averaged over triplicate experiments and presented with the standard error of mean (SEM). Peak areas were normalized by the peak area of the internal standard tramadol‐^13^CD_3_ and plotted relative to their observed peak area maximum.

In order to investigate the variation of the in vitro metabolic profile of *N*‐pyrrolidino fluetonitazene over time, *N*‐pyrrolidino fluetonitazene was incubated with pHLM for either 1, 2, 4 or 6 h (see Figure 5 and Figure S2).

**FIGURE 6:**
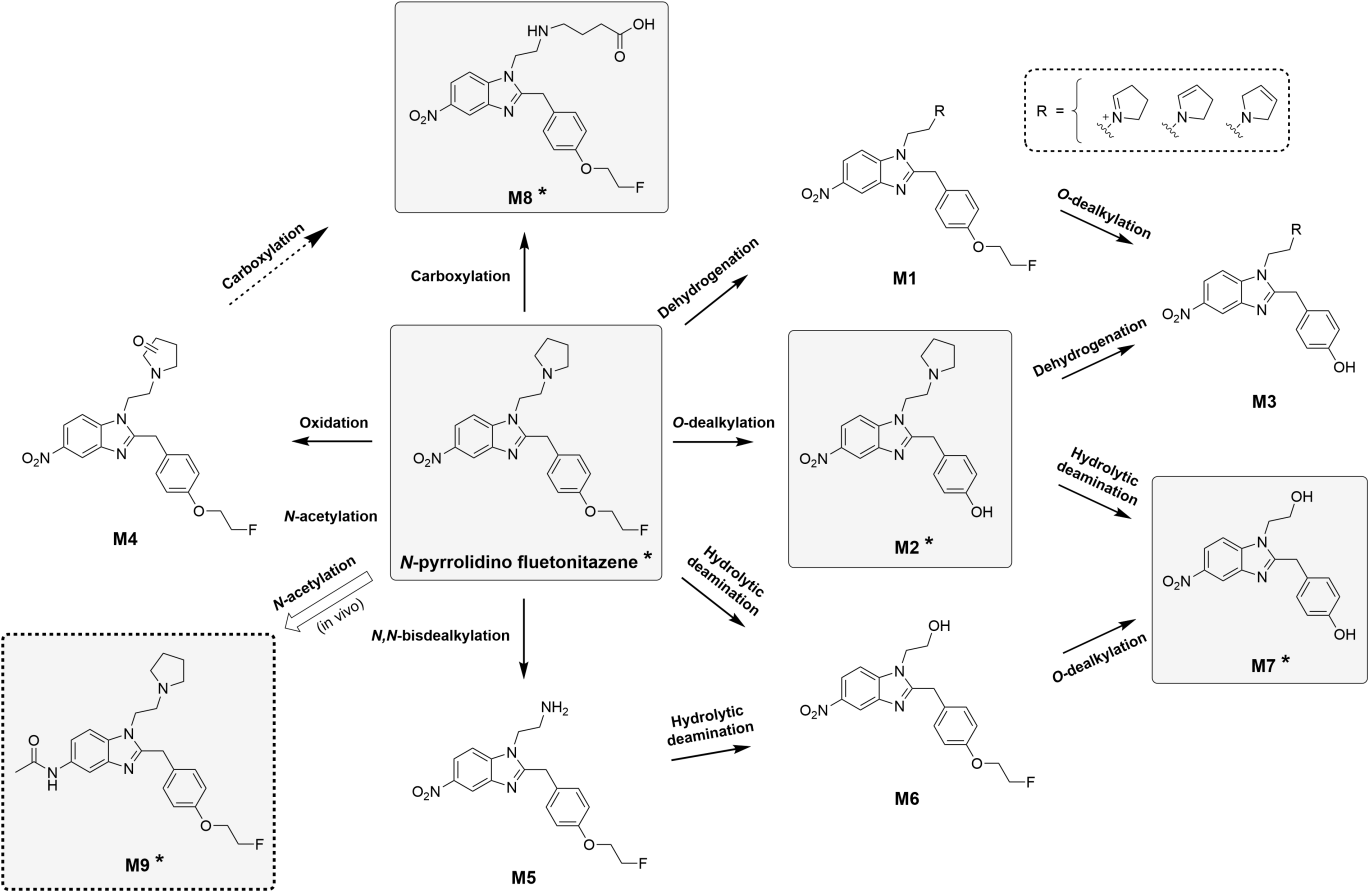
Proposed metabolic pathways of *N*‐pyrrolidino fluetonitazene. Metabolites labelled with an asterisk (*) were both found in vitro and in an authentic urine case sample. Metabolite **M9** in the grey dashed box was found in all three postmortem samples but not in vitro.

All four incubation times generated metabolites **M1**–**M8**. However, two distinct trends were observed with respect to the change in peak area over time in the extracted ion chromatograms (XIC). The first group of metabolites (**M1, M2, M5** and **M6**) showed a trend of decreasing peak areas as the incubation time increased (Figure 5A). The opposite behaviour was observed for the second group (**M3, M4, M7** and **M8**), where the metabolites reached their maximum observed peak area after 6 h incubation (Figure 5B). Metabolites of the first group all featured single biotransformations, whereas multiple biotransformations were found in the second group. The decrease of peak area in the first group over time therefore suggests further metabolism and hence transition to the second group where the metabolites accumulate. This can be observed for metabolite **M1** (hydrogenation) and metabolite **M2** (*O*‐dealkylation), both “intermediate” metabolites, which can both transform to metabolite **M3** (*O*‐dealkylation & dehydrogenation). The same pattern is found with metabolite **M6** (hydrolytic deamination), which is transformed further to metabolite **M7** (*O*‐dealkylation & hydrolytic deamination). Metabolite **M5** can undergo hydrolytic deamination, which potentially generates metabolite **M6** [33, 34]. In summary, observed metabolites accumulate over time unless they act as a substrate for subsequent biotransformations, which is illustrated in the proposed pathway in Figure 6.

### 3.3 Identification of In Vivo Metabolites

For the investigation of the in vivo metabolism of *N*‐pyrrolidino fluetonitazene, forensic case samples (femoral blood, heart blood and urine samples) were analysed. In the urine sample, the parent compound was only detectable in traces in a SWATH experiment (and not in the IDA experiment). SWATH enables detection of low‐abundance compounds due to its data‐independent acquisition strategy, but it results in more complex and less selective MS/MS spectra [35]. For reliable metabolite identification, IDA was preferred when available due to its higher spectral clarity, while SWATH was used as a complementary approach (when IDA was not available).

In the urine sample, three metabolites (**M2** (*O*‐dealkylation), **M7** (*O*‐dealkylation & hydrolytic deamination), **M8** (oxidative ring opening & carboxylation)) were also observed in vitro, as well as one novel metabolite **M9** (*N*‐acetylation) that was detected. The corresponding extracted ion chromatogram as well as the MS/MS spectra of the urine sample can be seen in Figures S3 and S5A–D. In the two blood samples, only one metabolite **M9** but not the parent compound *N*‐pyrrolidino fluetonitazene was detected (10‐times concentrated samples (compare Section 3.4)). The corresponding extracted ion chromatogram as well as the MS/MS spectra of one representative blood sample can be seen in Figures S4 and S5E.

**M2** and **M7** are *O*‐dealkylated and thus have lost the characteristic fluoroethyl moiety on the alkoxy benzyl side chain. This is a recurring challenge of nitazene detection in forensic case samples, as these metabolites are shared between all members of the *N*‐pyrrolidino class of nitazenes, and hence a conclusive identification of a distinct *N*‐pyrrolidino nitazene is not possible. Aligning with this, Kriikku et al. [8] reported on five cases involving *N*‐pyrrolidino fluetonitazene in Finland and recommended the *O*‐dealkylated metabolite (**M2**) for general detection of this class of nitazenes.

Metabolite **M8** (carboxylation to *N*‐butanoic acid) had a lower intensity with just over 2000 counts per second in the XIC. To our knowledge, this is the first characteristic metabolite of *N*‐pyrrolidino fluetonitazene reported in the literature. While the MS/MS spectrum of the compound features many intense fragmentation products of the *N*‐butanoic acid chain, which are not characteristic, fragment *m/z* 316.1090 (C_16_H_15_FN_3_O_3_^+^, −0.6 ppm, 17% relative intensity) does contain the fluoroethyl moiety. Metabolite **M8** thus allowed unambiguous identification of the parent compound in an authentic urine case sample. We therefore recommend the characteristic *N*‐butanoic acid metabolite **M8** for confirmation of *N*‐pyrrolidino fluetonitazene in forensic cases.

In both blood and urine, **M9** (5‐acetamido‐*N*‐pyrrolidino fluetodesnitazene) was the most abundant metabolite. It is formed by *N*‐acetylation of 5‐amino‐*N*‐pyrrolidino fluetodesnitazene (see Figure S5E), which is a commonly observed biotransformation of nitazene analogues [36, 37, 38]. However, in the present study, the intermediate 5‐amino‐*N*‐pyrrolidino fluetodesnitazene was not detected in the case samples. This may be due to efficient acetylation to **M9**, to the point where 5‐amino‐*N*‐pyrrolidino fluetodesnitazene is no longer detectable in the biological matrix. In a study of metonitazene, the authors reported a 30‐fold higher peak area of 5‐acetamido metodesnitazene compared to its amino counterpart in blood samples, suggesting high turnover [39]. This intermediate is formed via Phase I reduction of the 5‐nitro group on the benzimidazole ring, a metabolic pathway frequently reported for nitazene analogues and other nitroaryl drugs [40]. *N*‐acetylation is a Phase II reaction of aryl‐amino compounds mediated by *N*‐acetyltransferase enzymes (NAT 1 and NAT 2) [41], with polymorphisms resulting in interindividual differences in acetylation [42]. Hence, the absence of the intermediate 5‐amino‐*N*‐pyrrolidino fluetodesnitazene despite the presence of **M9** may indicate efficient acetylation of the amino metabolite, potentially associated with the metabolic phenotype of the deceased. Since *N*‐acetylation is a Phase II reaction, it is not surprising that in the present study this biotransformation was not observed in pHLM samples, since they can only form Phase I metabolites [13].

Based on the in vitro and in vivo results, a metabolic pathway of *N*‐pyrrolidino fluetonitazene was proposed (see Figure 6). Three out of four metabolites observed in the case samples were also detected in vitro. The most abundant metabolite **M9** was only observed in the postmortem samples.

### 3.4 Detection and Quantitative Analysis of *N*‐Pyrrolidino Fluetonitazene and Other Drugs in the Authentic Case Samples

The postmortem blood (femoral and cardiac blood) and urine samples were quantified for *N*‐pyrrolidino fluetonitazene with the described method. In the present case, *N*‐pyrrolidino fluetonitazene concentrations were at the lower limit of quantification (femoral blood: < 0.75 ng/mL; cardiac blood: 1 ng/mL). In the urine sample, *N*‐pyrrolidino fluetonitazene could not be quantified, even when concentrating the sample 10 times. The differences between femoral and cardiac blood can be attributed to postmortem redistribution. Recently, Kriikku et al. [8] investigated the concentration of *N*‐pyrrolidino fluetonitazene and isotonitazepyne in postmortem blood and urine samples from six different forensic cases. *N*‐pyrrolidino fluetonitazene was quantified in five of the cases in concentrations ranging from 0.97 to 10 ng/mL (median: 1.3 ng/mL) in blood and from 0.6 to > 100 ng/mL (median: 23 ng/mL) in urine samples.

Additionally, the general unknown screening as well as the confirmation procedures revealed the presence of ethyl alcohol (0.55 g/kg), delta‐9‐tetrahydrocannabinol (THC, < 0.75 ng/mL) and its metabolites 11‐hydroxy‐THC (1.9 ng/mL), 11‐nor‐9‐carboxy‐THC (23 ng/mL), high citalopram concentration (340 ng/mL), desmethylcitalopram (140 ng/mL) and a subtherapeutic concentration of lithium (1670 ng/mL) in femoral blood.

### 3.5 Evaluation of Utilized In Silico Predictions

GLORYx was used as an in silico model to predict potential metabolites of *N*‐pyrrolidino fluetonitazene. Based on *N*‐pyrrolidino fluetonitazene as input, GLORYx predicted 37 metabolites, 36 of which were phase I. When using its *O*‐dealkylated metabolite (**M2**), GLORYx predicted 33 metabolites, 30 of which were phase I. The software predominantly suggested aliphatic hydroxylations in various positions, as well as *O*‐dealkylation, deamination, dehydrogenation and nitro reduction. Out of the 36 phase I predictions of the parent compound, six metabolites were observed directly in the in vitro metabolism experiments when including regioisomers of metabolite **M1** (dehydrogenation of the pyrrolidine ring). Phase II metabolism of the parent compound included glucuronidation of the benzimidazole core in the 3‐position, with an assigned priority score of 0.06. In contrast, glucuronidation and sulfation of the *O*‐dealkylated metabolite (**M2**) yielded a priority score of 0.98 and 0.95 respectively, twice as high as any phase I metabolite. The model therefore predicts a high likelihood of phase II metabolite formation at the 4′‐position after the fluoroethyl side chain has been cleaved.

GLORYx was able to predict six of the eight metabolites **M1**–**M8** detected in the in vitro experiments, highlighting its usefulness as a versatile prediction tool. It was also able to predict **M9** as a subsequent metabolite when 5‐amino‐N‐pyrrolidino fluetonitazene was used as an intermediate‐input (prediction score of 0.85). Since we did not observe 5‐amino‐N‐pyrrolidino fluetonitazene, **M9** was identified via the analysis of literature on nitazene analogues. However, the software was unable to predict *N,N*‐bisdealkylation resulting in a primary amine (**M5**) or ring opening upon carboxylation resulting in the *N*‐butanoic acid metabolite (**M8**). Furthermore, the model prominently predicted hydroxylation in various positions, which was not observed in the in vitro experiments. In fact, the highest‐ranking result was aliphatic hydroxylation of the fluoroethyl side chain (priority score 0.75). Given that hydroxylation metabolites are commonly reported in vitro pHLM experiments of other nitazene analogues [25, 27, 28], we tentatively suggest that the fluoro substituent inhibits hydroxylation on the fluoroethyl moiety, which is therefore not accurately reflected in the in silico model.

### 3.6 MOR Affinity of *N*‐Pyrrolidino Fluetonitazene

**FIGURE 7:**
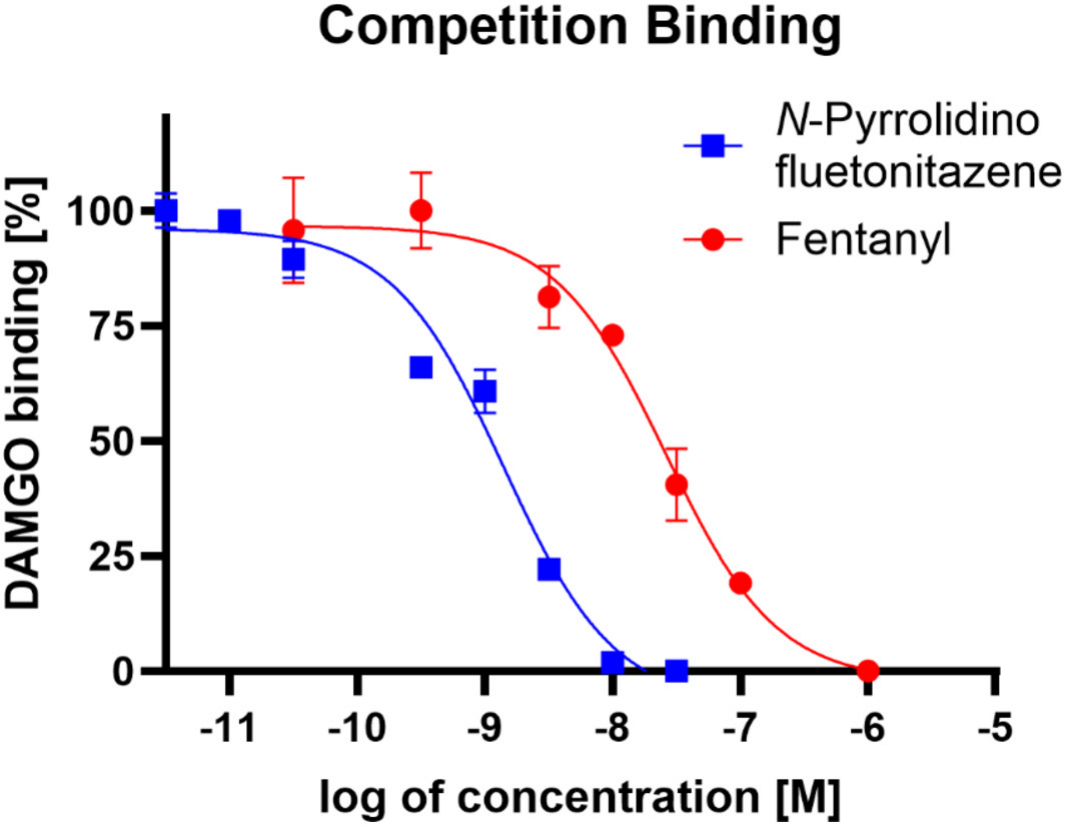
Concentration‐displacement curves of *N*‐pyrrolidino fluetonitazene and the reference compound fentanyl at the hMOR derived from DAMGO mediated in vitro receptor affinity assay upon concentration‐dependent stimulation data is depicted as mean ± standard error of mean (SEM).

In the present study, the MOR affinity of *N*‐pyrrolidino fluetonitazene was assessed using an LC–MS/MS‐based competition binding assay with DAMGO. Compared to the reference compound fentanyl, which was taken along on every single assay plate as a quality control, *N*‐pyrrolidino fluetonitazene depicted a MOR affinity almost 20 times stronger with a K_i_ value of 0.166 nM (see Table 3 and Figure 7).

**TABLE 3:** Binding affinities (K_i_, 95% confidence interval [CI]) of *N*‐pyrrolidino fluetonitazene and the reference compound fentanyl at the human MOR determined using an LC–MS/MS‐based competition binding assay. Additionally, published K_i_ values from radioligand binding assays at the human MOR are provided for comparison (reported as mean ± SD where available). Literature values for fentanyl were restricted to studies using human MOR. SD‐standard deviation.

Substrate	K_i_ (95% CI) [nM]	K_i_ literature radioligand assays [nM]
*N*‐pyrrolidino fluetonitazene	0.166 (0.115–0.237)	0.342 ± 0.014 [43]
Fentanyl	3.090 (1.770–5.360)	2.9 [44] 3.3 [45] 5.23 [46] 1.88 ± 0.23 [43] Mean ± SD: 3.33 ± 1.21

In a MOR radioligand [^3^H]DAMGO competition binding assay, *N*‐pyrrolidino fluetonitazene was binding 3.7 to 6.4 times stronger, compared to the reference compounds DAMGO (K_i_ = 1.26 ± 0.13 nM), fentanyl (K_i_ = 1.88 ± 0.23 nM) and morphine (K_i_ = 2.18 ± 0.20 nM), with a K_i_ in the subnanomolar range (0.342 ± 0.0014 nM) [43]. In the same study, its KOR and DOR affinities were also assessed ([^3^H]U69,593 and [^3^H]DPDPE competition binding assay) and *N*‐pyrrolidino fluetonitazene showed a clear bias towards MOR over KOR (K_i_ = 1110 ± 260 nM) and DOR (K_i_ = 153 ± 12 nM). Furthermore, the MOR, KOR and DOR receptor activities were assessed using [^35^S]GTPγS functional assays and *N*‐pyrrolidino fluetonitazene (EC_50_ = 2.40 ± 0.25 nM) had MOR potencies 12.7–7.4 times higher, compared to the reference compounds DAMGO (EC_50_ = 17.8 ± 1.4 nM), fentanyl (EC_50_ = 30.4 ± 1.2 nM) and morphine (EC_50_ = 22.3 ± 2.2 nM).

While radioligand‐based receptor affinity assays have been primarily used in the past, LC–MS/MS‐based assays have several advantages, as long as the same cold ligands are used. Limitations of radioligand‐based assays include the generation of radioactive waste with its environmental implications, relatively long data acquisition times, higher operational costs (including the maintenance of an isotopic laboratory), health and safety risks and the need for specialized licensing and regulatory compliance [47, 48]. A comparison between K_i_ values reported in the literature and our results was performed for fentanyl (only data using [^3^H]DAMGO at the human MOR receptor were considered), with our data aligning with previous publications (see Table 3). Our results confirm that the LC–MS/MS‐based MOR affinity assay is suitable for assessing *N*‐pyrrolidino fluetonitazene and most likely other nitazenes.

### 3.7 Limitations of This Study

Since this sample is the only known case involving *N*‐pyrrolidino fluetonitazene in Switzerland at the time of writing, more cases are needed to find reliable and robust metabolic patterns in forensic samples. Furthermore, in the present study, only postmortem samples from a single timepoint were available, while metabolite profiles may shift during the course of elimination. In addition, interindividual differences in metabolism, including enzyme polymorphism affecting the formation of metabolites such as **M9**, are to be expected.

## 4 Conclusion

In the present study, eight different metabolites of *N*‐pyrrolidino fluetonitazene were identified in vitro in pHLM. In the urine sample, three metabolites (**M2** (*O*‐dealkylation), **M7** (*O*‐dealkylation & hydrolytic deamination), **M8** (oxidative ring opening & carboxylation)) were also observed in vitro, as well as one novel metabolite **M9** (*N*‐acetylation) that was detected. However, in the two blood samples, only **M9** was present.

The parent compound was quantifiable using LC–MS/MS in the two blood samples but could only be detected in the urine sample using LC‐HR‐MS/MS. Concentrations found in the two blood samples were aligned with data from five *N*‐pyrrolidino fluetonitazene intoxication cases in Finland and were in the range of < 0.75 to 1 ng/mL. In vitro, the most abundant metabolites were **M1‐M3** formed by dehydrogenation, *O*‐dealkylation and their combination. In the postmortem case samples, **M9** was the most abundant metabolite followed by **M2** (4′‐OH‐nitazepyne), which is a metabolite common to all nitazepyne‐type substances and a useful marker compound in urine screenings [8]. For the confirmation of *N*‐pyrrolidino fluetonitazene, we recommend including **M8** and **M9** as marker metabolites since they are specific for this nitazene. The observed metabolic pathways are consistent with previously reported metabolic pathways of nitazenes. In addition, our results suggest that fluoro substitution on the ethoxy moiety inhibits hydroxylation on the oxygen‐containing side chain.

Furthermore, we demonstrated that the LC–MS/MS‐based MOR receptor affinity assay yields valid results for *N*‐pyrrolidino fluetonitazene that are consistent with those obtained using established radioligand‐based methods.

In the absence of any other cause, the death was attributed to an acute intoxication with *N*‐pyrrolidino fluetonitazene and ethanol, in presence of the therapeutic drugs citalopram and lithium.

The present forensic case underlines the particular value of metabolite detection in cases where the parent compound is present at very low concentrations as it allows confirmation of consumption and identification of the parent compound.

## Author Contributions

**Severin Zemp:** investigation, data curation, data analysis, visualization, writing – original draft. **Gaia Alluisetti:** investigation, data curation, visualization, writing – review and editing. **Wolfgang Weinmann:** writing – review and editing. **Bettina Schrag:** writing – review and editing. **Frank Sporkert:** investigation, data curation, writing – review and editing. **Maurine Leclerc:** writing – review and editing. **Katharina Elisabeth Grafinger:** conceptualization, methodology, project administration, supervision, writing – original draft.

## Funding

This work was supported by the Swiss National Science Foundation (SNF_10000358).

## Conflicts of Interest

The authors declare no conflicts of interest.

## Supporting information


**Table S1:** Retention times, chemical formulas, ring and double bond equivalents (RDB), mass errors and intensities for compounds (extracted ion chromatogram peak height) and fragments (MS/MS spectrum signal height) of N‐pyrrolidino fluetonitazene and its identified. All values are averaged over triplicate experiments. Normalized intensities of fragment ions relative to the most intense peak are given as subscript. (*) ions represent fragments containing the characteristic fluoroethyl moiety. Compounds with peak heights of fewer than 1000 counts per second were not included, except for metabolite M8, which was also detected in vivo.
**Figure S1:** Extracted ion chromatogram (XIC) of the in vitro metabolites of *N*‐pyrrolidino fluetonitazene after 1 h of incubation with pooled human liver microsomes (pHLM).
**Figure S2:** Relative intensities of the parent compound and metabolites M1–M8 after 1, 2, 4 and 6 h incubation in pooled human liver microsomes (pHLM). All values are averaged over triplicate experiments.
**Figure S3:** Extracted ion chromatogram (XIC) of the in vivo metabolites of *N*‐pyrrolidino fluetonitazene in an authentic urine case sample.
**Figure S4:** Extracted ion chromatogram (XIC) of the in vivo metabolites of *N*‐pyrrolidino fluetonitazene in an authentic blood case sample.
**Figure S5:** Data independent acquisition (SWATH) and information dependent acquisition (IDA) MS/MS spectra of (A) N‐pyrrolidino fluetonitazene and (B–D) in vivo metabolites in a urine case sample and (E) in all three postmortem sample types. The parent compound underwent (B) O‐dealkylation (**M2**), (C) O‐dealkylation and hydrolytic deamination (**M7**) and (D) pyrrolidine ring opening via carboxylation (**M8**). (E) metabolite **M9** was formed by *N*‐acetylation. Fragmentation of selected precursor ions was conducted with a ce of 35 eV and a CES of ±15 eV.

## Data Availability

The data that support the findings of this study are available in the Supporting Information of this article. Any additional data are available from the authors upon reasonable request.
