# Supplementary material for: Investigation of the In Vitro and In Vivo Metabolism and μ‐Opioid Receptor Affinity of the Nitazene N‐Pyrrolidino Fluetonitazene
Source: Drug Test Anal. 2026 Jun 2;18(8):1097–113. doi: 10.1002/dta.70095 (PMC13432772; doi:10.1002/dta.70095)
Supplement: Supplementary file 1 — Table S1: Retention times, chemical formulas, ring and double bond equivalents (RDB), mass errors and intensities for compounds (extracted ion chromatogram peak height) and fragments (MS/MS spectrum signal height) of N‐pyrrolidino fluetonitazene and its identified. All values are averaged over triplicate experiments. Normalized intensities of fragment ions relative to the most intense peak are given as subscript. (*) ions represent fragments containing the characteristic fluoroethyl moiety. Compounds with peak heights of fewer than 1000 counts per second were not included, except for metabolite M8, which was also detected in vivo. Figure S1: Extracted ion chromatogram (XIC) of the in vitro metabolites of N‐pyrrolidino fluetonitazene after 1 h of incubation with pooled human liver microsomes (pHLM). Figure S2: Relative intensities of the parent compound and metabolites M1–M8 after 1, 2, 4 and 6 h incubation in pooled human liver microsomes (pHLM). All values are averaged over triplicate experiments. Figure S3: Extracted ion chromatogram (XIC) of the in vivo metabolites of N‐pyrrolidino fluetonitazene in an authentic urine case sample. Figure S4: Extracted ion chromatogram (XIC) of the in vivo metabolites of N‐pyrrolidino fluetonitazene in an authentic blood case sample. Figure S5: Data independent acquisition (SWATH) and information dependent acquisition (IDA) MS/MS spectra of (A) N‐pyrrolidino fluetonitazene and (B–D) in vivo metabolites in a urine case sample and (E) in all three postmortem sample types. The parent compound underwent (B) O‐dealkylation (M2), (C) O‐dealkylation and hydrolytic deamination (M7) and (D) pyrrolidine ring opening via carboxylation (M8). (E) metabolite M9 was formed by N‐acetylation. Fragmentation of selected precursor ions was conducted with a ce of 35 eV and a CES of ±15 eV. [file DTA-18-1097-s001.docx]

**SUPPORTING INFORMATION**

**Investigation of the *in vitro* and *in vivo* metabolism and μ-opioid receptor affinity of the nitazene *N*-pyrrolidino fluetonitazene**

Severin Zemp^1^, Gaia Alluisetti^1,2^, Wolfgang Weinmann^1^, Bettina Schrag^3^, Frank Sporkert^4^, Maurine Leclerc^4^, Katharina Elisabeth Grafinger^1,§^,

^1^ Institute of Forensic Medicine Bern, Forensic Toxicology and Chemistry, University of Bern, Murtenstrasse 26 Bern, Switzerland

^2^ Graduate School for Cellular and Biomedical Sciences, University of Bern, Switzerland

^3^ ICH Central Institute, Department of Forensic Medicine, Avenue du Grand-Champsec 86, 1951 Sion, Switzerland

^4^ University Centre of Legal Medicine Lausanne-Geneva, Forensic Toxicology and Chemistry Unit, Chemin de la Vulliette 4, 1000 Lausanne 25, Switzerland

^§^Corresponding author: Katharina Elisabeth Grafinger, Institute of Forensic Medicine Bern, Forensic Toxicology and Chemistry, Murtenstrasse 26, 3008 Bern, Switzerland, Katharina.grafinger@irm.unibe.ch

**Orcid:**

Katharina Elisabeth Grafinger <https://orcid.org/0000-0002-3647-7455>

Gaia Alluisetti <https://orcid.org/0009-0002-6098-1739>

Wolfgang Weinmann <https://orcid.org/0000-0001-8659-1304>

**Table SI 1:** Retention times, chemical formulas, ring and double bond equivalents (RDB), mass errors, and intensities for compounds (extracted ion chromatogram peak height) and fragments (MS/MS spectrum signal height) of N-pyrrolidino fluetonitazene and its identified. All values are averaged over triplicate experiments. Normalized intensities of fragment ions relative to the most intense peak are given as subscript. (*) ions represent fragments containing the characteristic fluoroethyl moiety. Compounds with peak heights of fewer than 1000 counts per second were not included, except for metabolite M8, which was also detected *in vivo*.

| **Compound Name** | **Biotransformation** | **Matrix** | **RT [min]** | **Chemical Formula [M+H]^+^** | **RDB** | **Theoretical m/z [Da]** | **Measured m/z [Da]** | **Error [ppm]** | **Intensity [cps]** |
| --- | --- | --- | --- | --- | --- | --- | --- | --- | --- |
| **N-pyrrolidino** | Parent drug | pHLM | 16.61 | C_22_H_26_FN_4_O_3_^+^ | 12 | 413.19835 | 413.1996 *** | 3.1 | 1.3 × 10^5^ |
| **fluetonitazene** |  | urine |  | C_6_H_12_N^+^ | 2 | 98.09643 | 98.0968 | 3.4 | 4.5 × 10^4^***_100_*** |
|  |  |  |  | C_9_H_10_FO^+^ | 5 | 153.07102 | 153.0709 * | -0.6 | 2.1 × 10^3^***_5_*** |
|  |  |  |  | C_7_H_7_O^+^ | 5 | 107.04914 | 107.0490 | -1.6 | 1.6 × 10^3^***_4_*** |
|  |  |  |  | C_3_H_6_N^+^ | 2 | 56.04948 | 56.0494 | -1.4 | 1.6 × 10^3^***_4_*** |
|  |  |  |  | C_18_H_17_FN_2_O^•+^ | 11.5 | 296.13194 | 296.1323 * | 1.2 | 1.9 × 10^2^***_< 1_*** |
|  |  |  |  | C_18_H_17_FN_3_O_3_^+^ | 12 | 342.12485 | 342.1248 * | -0.1 | 1.1 × 10^2^***_< 1_*** |
|  |  |  |  | C_18_H_17_FN_2_O_3_^•+^ | 11.5 | 328.12177 | 328.1229 * | 3.3 | 1.1 × 10^2^***_< 1_*** |
|  |  |  |  | C_14_H_17_N_4_O_2_^+^ | 9 | 273.13460 | 273.1355 | 3.2 | 9.2 × 10^1^***_<1_*** |
| **M1** | Oxidation | pHLM | 15.61 | C_22_H_24_FN_4_O_3_^+^ | 13 | 411.18270 | 411.1835 * | 2 | 1.8 × 10^4^ |
|  | (- 2H) |  |  | C_6_H_10_N^+^ | 3 | 96.08078 | 96.0806 | -1.9 | 2.4 × 10^3^***_100_*** |
|  |  |  |  | C_9_H_10_FO^+^ | 5 | 153.07102 | 153.0709 * | -0.8 | 1.1 × 10^3^***_45_*** |
|  |  |  |  | C_7_H_7_O^+^ | 5 | 107.04914 | 107.0488 | -2.9 | 8.4 × 10^2^***_35_*** |
|  |  |  |  | C_18_H_17_FN_2_O^•+^ | 11.5 | 296.13194 | 296.1326 * | 2.1 | 4.1 × 10^2^***_17_*** |
|  |  |  |  | C_16_H_13_FN_3_O_3_^+^ | 12 | 314.09355 | 314.0942 * | 2 | 2.1 × 10^2^***_9_*** |
|  |  |  |  | C_18_H_17_FN_2_O_3_^•+^ | 11.5 | 328.12177 | 328.1224 * | 1.8 | 2.0 × 10^2^***_8_*** |
|  |  |  |  | C_16_H_13_FN_2_O^•+^ | 11.5 | 268.10064 | 268.1018 * | 4.2 | 1.5 × 10^2^***_6_*** |
|  |  |  |  | C_13_H_14_N_4_O_2_^•+^ | 9.5 | 258.11113 | 258.1113 | 0.7 | 1.4 × 10^2^***_6_*** |
|  |  |  |  | C_5_H_9_^+^ | 2 | 69.06988 | 69.0696 | -3.6 | 1.1 × 10^2^***_4_*** |
|  |  |  |  | C_18_H_17_FN_3_O_3_^+^ | 12 | 342.12485 | 342.1253 * | 1.3 | 1.1 × 10^2^***_5_*** |
|  |  |  |  | C_16_H_13_FN_2_O_3_^•+^ | 11.5 | 300.09047 | 300.0913 * | 2.8 | 7.7 × 10^1^***_3_*** |
| **M2** | *O*-dealkylation | pHLM | 10.04 | C_20_H_23_N_4_O_3_^+^ | 12 | 367.17647 | 367.1766 | 0.3 | 5.5 × 10^3^ |
|  | (- C_2_H_3_F) | urine |  | C_6_H_12_N^+^ | 2 | 98.09643 | 98.0962 | -2.7 | 2.2 × 10^3^***_100_*** |
|  |  |  |  | C_7_H_7_O^+^ | 5 | 107.04914 | 107.0487 | -3.8 | 2.7 × 10^2^***_12_*** |
|  |  |  |  | C_3_H_6_N^+^ | 2 | 56.04948 | 56.0492 | -4.4 | 1.6 × 10^2^***_7_*** |
|  |  |  |  | C_16_H_14_N_2_O^•+^ | 11.5 | 250.11006 | 250.1106 | 2.2 | 3.8 × 10^1^***_2_*** |
|  |  |  |  | C_16_H_14_N_2_O_3_^•+^ | 11.5 | 282.09989 | 282.1002 | 1.1 | 2.1 × 10^1^***_1_*** |
|  |  |  |  | C_16_H_14_N_3_O_3_^+^ | 12 | 296.10297 | 296.1006 | -8.1 | 9.3 × 10^0^***_< 1_*** |
| **M3** | *O*-dealkylation & | pHLM | 7.38 | C_20_H_21_N_4_O_3_^+^ | 13 | 365.16082 | 365.1618 | 2.7 | 6.5 × 10^3^ |
|  | Oxidation |  |  | C_6_H_10_N^+^ | 3 | 96.08078 | 96.0805 | -2.6 | 9.6 × 10^2^***_100_*** |
|  | (- C_2_H_3_F, - 2H) |  |  | C_7_H_7_O^+^ | 5 | 107.04914 | 107.0488 | -2.9 | 5.2 × 10^2^***_54_*** |
|  |  |  |  | C_16_H_14_N_2_O^•+^ | 11.5 | 250.11006 | 250.1105 | 1.8 | 3.0 × 10^2^***_31_*** |
|  |  |  |  | C_14_H_10_N_2_O^•+^ | 11.5 | 222.07876 | 222.0791 | 1.7 | 1.5 × 10^2^***_16_*** |
|  |  |  |  | C_16_H_14_N_2_O_3_^•+^ | 11.5 | 282.09989 | 282.1003 | 1.5 | 1.3 × 10^2^***_14_*** |
|  |  |  |  | C_13_H_15_N_4_O_2_^+^ | 9 | 259.11895 | 259.1192 | 1.1 | 1.3 × 10^2^***_14_*** |
|  |  |  |  | C_14_H_10_N_3_O_3_^+^ | 12 | 268.07167 | 268.0721 | 1.5 | 8.0 × 10^1^***_8_*** |
|  |  |  |  | C_13_H_14_N_4_O_2_^•+^ | 9.5 | 258.11113 | 258.1116 | 1.7 | 6.3 × 10^1^***_7_*** |
|  |  |  |  | C_14_H_10_N_2_O_3_^•+^ | 11.5 | 254.06859 | 254.0692 | 2.5 | 6.1 × 10^1^***_6_*** |
|  |  |  |  | C_16_H_14_N_3_O_3_^+^ | 12 | 296.10297 | 296.1028 | -0.6 | 3.9 × 10^1^***_4_*** |
| **M4** | Hydroxylation & | pHLM | 23.96 | C_22_H_24_FN_4_O_4_^+^ | 13 | 427.17761 | 427.1789 * | 2.9 | 5.7 × 10^3^ |
|  | Oxidation |  |  | C_6_H_10_NO^+^ | 3 | 112.07569 | 112.0755 | -1.4 | 4.0 × 10^3^***_100_*** |
|  | (+ O, - 2H) |  |  | C_4_H_5_O^+^ | 3 | 69.03349 | 69.0335 | -0.3 | 1.5 × 10^2^***_4_*** |
|  |  |  |  | C_5_H_10_N^+^ | 2 | 84.08078 | 84.0804 | -4.1 | 9.7 × 10^1^***_2_*** |
| **M5** | *N,N*-bisdealkylation | pHLM | 14.30 | C_18_H_20_FN_4_O_3_^+^ | 11 | 359.1514 | 359.1520 * | 1.8 | 1.9 × 10^3^ |
|  | (- C_4_H_6_) |  |  | C_16_H_15_FN_3_O_3_^+^ | 11 | 316.1092 | 316.1098 * | 2 | 5.8 × 10^2^***_100_*** |
|  |  |  |  | C_8_H_6_N_3_O_2_^+^ | 8 | 176.04545 | 176.0454 | -0.3 | 2.6 × 10^2^***_44_*** |
|  |  |  |  | C_16_H_15_FN_2_O^•+^ | 10.5 | 270.11629 | 270.1163 * | 0.2 | 1.9 × 10^2^***_33_*** |
|  |  |  |  | C_16_H_15_FN_2_O_3_^•+^ | 10.5 | 302.10612 | 302.1071 * | 3.2 | 1.3 × 10^2^***_21_*** |
|  |  |  |  | C_8_H_6_N_2_^•+^ | 7.5 | 130.05255 | 130.0523 | -1.7 | 7.5 × 10^1^***_13_*** |
|  |  |  |  | C_8_H_6_N_2_O^•+^ | 7.5 | 146.04746 | 146.0471 | -2.5 | 5.5 × 10^1^***_9_*** |
|  |  |  |  | C_7_H_6_N_2_^•+^ | 6.5 | 118.05255 | 118.0522 | -2.7 | 5.0 × 10^1^***_9_*** |
|  |  |  |  | C_9_H_10_FO^+^ | 5 | 153.07102 | 153.0712 * | 1.2 | 4.0 × 10^1^***_7_*** |
|  |  |  |  | C_7_H_7_O^+^ | 5 | 107.04914 | 107.0491 | -0.4 | 3.8 × 10^1^***_7_*** |
| **M6** | Oxidative deamination | pHLM | 20.16 | C_18_H_19_FN_3_O_4_^+^ | 11 | 360.13541 | 360.1360 * | 1.6 | 1.2 × 10^3^ |
|  | (- C_4_H_7_N, + O) |  |  | C_9_H_9_N_2_^+^ | 7 | 145.07602 | 145.0757 | -2.4 | 2.6 × 10^2^***_77_*** |
|  |  |  |  | C_18_H_19_FN_2_O_2_^•+^ | 10.5 | 314.14251 | 314.1430 * | 1.5 | 2.2 × 10^2^***_67_*** |
|  |  |  |  | C_18_H_19_FN_2_O_4_^•+^ | 10.5 | 346.13234 | 346.1324 * | 0.2 | 1.4 × 10^2^***_42_*** |
|  |  |  |  | C_7_H_7_O^+^ | 5 | 107.04914 | 107.0485 | -5.7 | 8.8 × 10^1^***_26_*** |
|  |  |  |  | C_16_H_15_FN_2_O^•+^ | 10.5 | 270.11629 | 270.1168 * | 1.8 | 7.3 × 10^1^***_22_*** |
|  |  |  |  | C_16_H_15_FN_3_O_3_^+^ | 11 | 316.10920 | 316.1092 * | 0.0 | 7.0 × 10^1^***_21_*** |
|  |  |  |  | C_8_H_6_N_3_O_2_^+^ | 8 | 176.04545 | 176.0455 | 0.1 | 6.5 × 10^1^***_19_*** |
|  |  |  |  | C_9_H_10_FO^+^ | 5 | 153.07102 | 153.0711 * | 0.3 | 5.8 × 10^1^***_17_*** |
|  |  |  |  | C_8_H_6_N_2_^•+^ | 7.5 | 130.05255 | 130.0533 | 5.8 | 5.7 × 10^1^***_17_*** |
|  |  |  |  | C_10_H_10_N_2_O^•+^ | 7.5 | 174.07876 | 174.0787 | -0.5 | 5.2 × 10^1^***_16_*** |
|  |  |  |  | C_16_H_15_FN_2_O_3_^•+^ | 10.5 | 302.10612 | 302.1062 * | 0.3 | 4.6 × 10^1^***_14_*** |
| **M7** | *O*-dealkylation & | pHLM | 11.42 | C_16_H_16_N_3_O_4_^+^ | 11 | 314.11353 | 314.1145 | 3.0 | 1.1 × 10^3^ |
|  | oxidative deamination | urine |  | C_16_H_16_N_2_O_2_^•+^ | 10.5 | 268.12063 | 268.1211 | 1.9 | 2.2 × 10^2^***_100_*** |
|  | (- C_6_H_10_FN, + O) |  |  | C_9_H_9_N_2_^+^ | 7 | 145.07602 | 145.0759 | -0.8 | 2.0 × 10^2^***_93_*** |
|  |  |  |  | C_14_H_12_N_2_O^•+^ | 10.5 | 224.09441 | 224.0943 | -0.3 | 1.3 × 10^2^***_58_*** |
|  |  |  |  | C_16_H_16_N_2_O_4_^•+^ | 10.5 | 300.11046 | 300.1108 | 1.2 | 1.2 × 10^2^***_55_*** |
|  |  |  |  | C_8_H_6_N_2_^•+^ | 7.5 | 130.05255 | 130.0522 | -2.4 | 9.3 × 10^1^***_43_*** |
|  |  |  |  | C_7_H_7_O^+^ | 5 | 107.04914 | 107.0487 | -3.8 | 8.1 × 10^1^***_38_*** |
|  |  |  |  | C_14_H_12_N_2_O_3_^•+^ | 10.5 | 256.08424 | 256.0844 | 0.6 | 6.1 × 10^1^***_28_*** |
|  |  |  |  | C_14_H_12_N_3_O_3_^+^ | 11 | 270.08732 | 270.0879 | 2.3 | 4.3 × 10^1^***_20_*** |
|  |  |  |  | C_8_H_6_N_3_O_2_^+^ | 8 | 176.04545 | 176.0453 | -1.0 | 4.1 × 10^1^***_19_*** |
|  |  |  |  | C_10_H_10_N_2_O^•+^ | 7.5 | 174.07876 | 174.0785 | -1.3 | 3.4 × 10^1^***_16_*** |
| **M8** | Carboxylation to | pHLM | 15.91 | C_22_H_26_FN_4_O_5_^+^ | 12 | 445.18817 | 445.1886 * | 1 | 7.5 × 10^2^ |
|  | *N*-butanoic acid | urine |  | C_6_H_12_NO_2_^+^ | 2 | 130.08626 | 130.0858 | -3.3 | 3.3 × 10^2^***_100_*** |
|  | (+ 2O) |  |  | C_6_H_10_NO^+^ | 3 | 112.07569 | 112.0753 | -3.8 | 1.4 × 10^2^***_42_*** |
|  |  |  |  | C_4_H_7_O_2_^+^ | 2 | 87.04406 | 87.0437 | -4.1 | 7.8 × 10^1^***_23_*** |
|  |  |  |  | C_16_H_15_FN_3_O_3_^+^ | 11 | 316.1092 | 316.1092 * | -0.1 | 5.7 × 10^1^***_17_*** |
|  |  |  |  | C_5_H_10_N^+^ | 2 | 84.08078 | 84.0804 | -4.5 | 3.2 × 10^1^***_10_*** |
|  |  |  |  | C_4_H_5_O^+^ | 3 | 69.03349 | 69.0334 | -0.8 | 3.0 × 10^1^***_9_*** |
|  |  |  |  | C_8_H_6_N_3_O_2_^+^ | 8 | 176.04545 | 176.0458 | 2 | 1.6 × 10^1^***_5_*** |
| **M9** | *N*-acetylation | urine | 7.73 | C_24_H_30_FN_4_O_2_^+^ | 12 | 425.23473 | 425.2342 *** | -1.3 | 1.9 × 10^4^ |
|  | (- O, + C_2_H_4_) | femoral blood |  | C_6_H_12_N^+^ | 2 | 98.09643 | 98.0961 | -3.4 | 1.0 × 10^4^***_100_*** |
|  |  | heart blood |  | C_9_H_10_FO^+^ | 5 | 153.07102 | 153.0704 * | -4.1 | 6.6 × 10^2^***_6_*** |
|  |  |  |  | C_7_H_7_O^+^ | 5 | 107.04914 | 107.0480 | -10.7 | 3.4 × 10^2^***_3_*** |
|  |  |  |  | C_3_H_6_N^+^ | 2 | 56.04948 | 56.0494 | -1.4 | 2.9 × 10^2^***_3_*** |


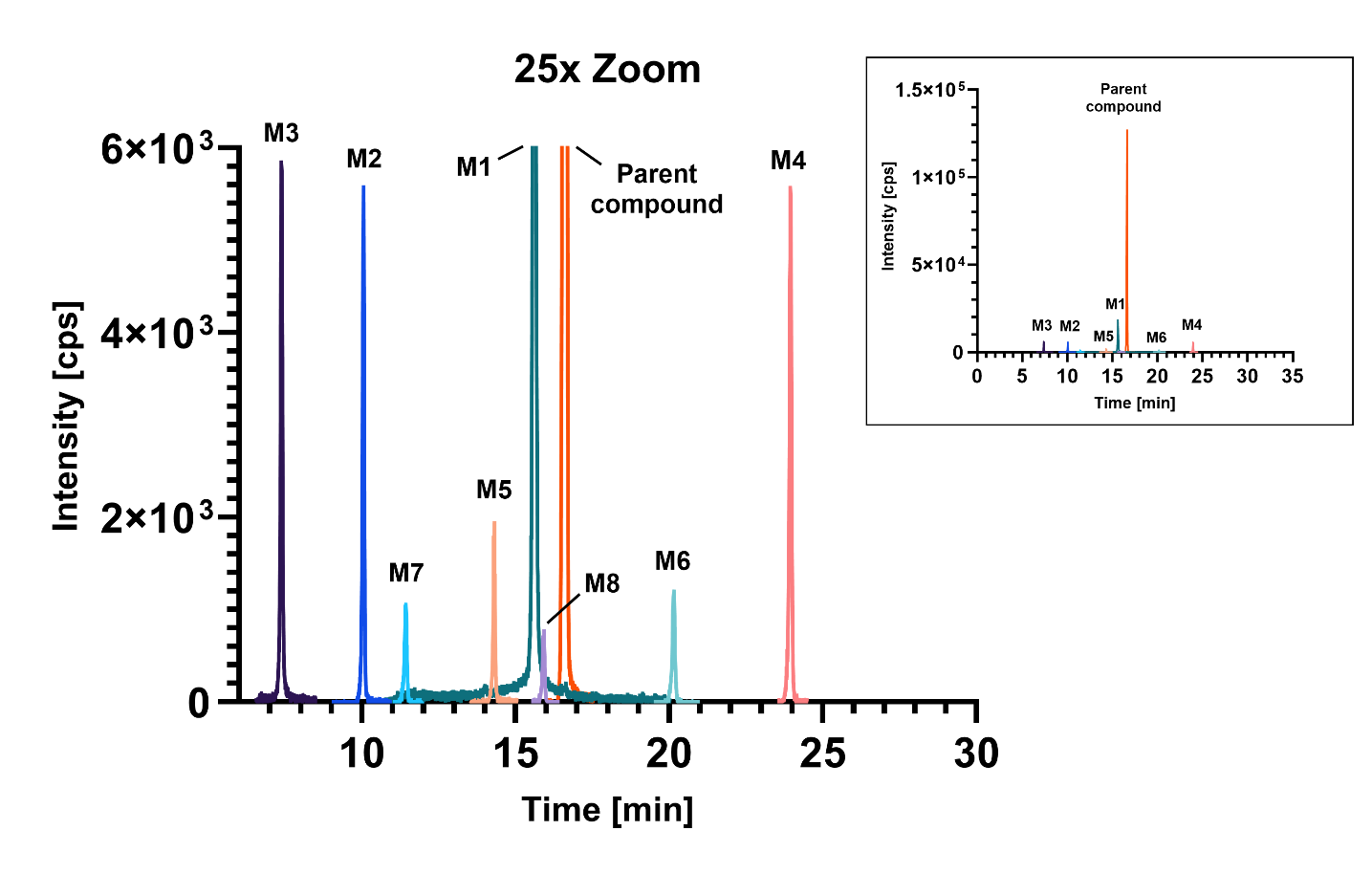


**Figure SI 1:** Extracted ion chromatogram (XIC) of the in vitro metabolites of N-pyrrolidino fluetonitazene after 1 hour of incubation with pooled human liver microsomes (pHLM).

**
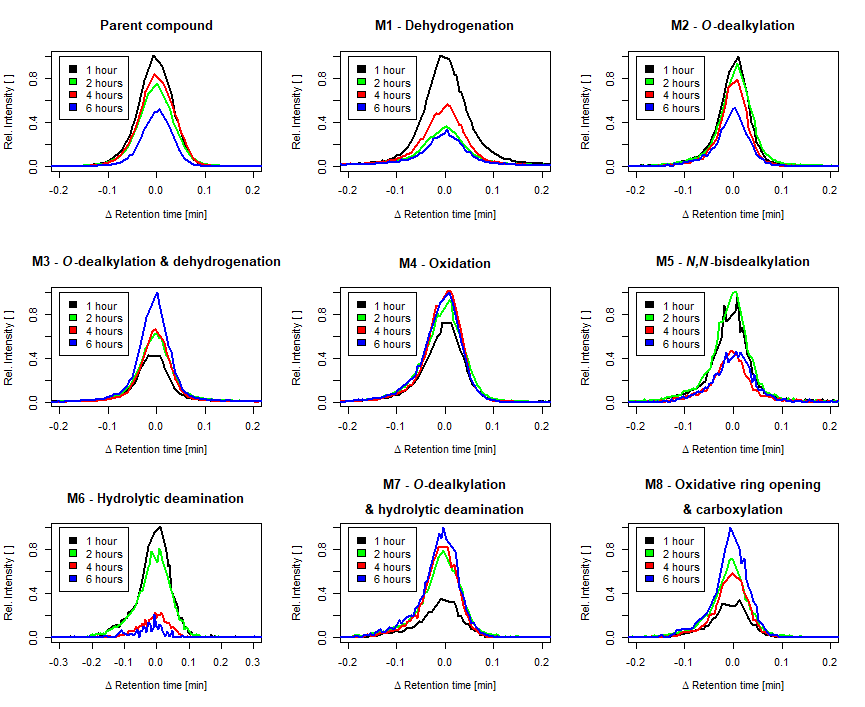
**

**Figure SI2**: Relative intensities of the parent compound and metabolites M1-M8 after 1, 2, 4 and 6 hours incubation in pooled human liver microsomes (pHLM). All values are averaged over triplicate experiments.


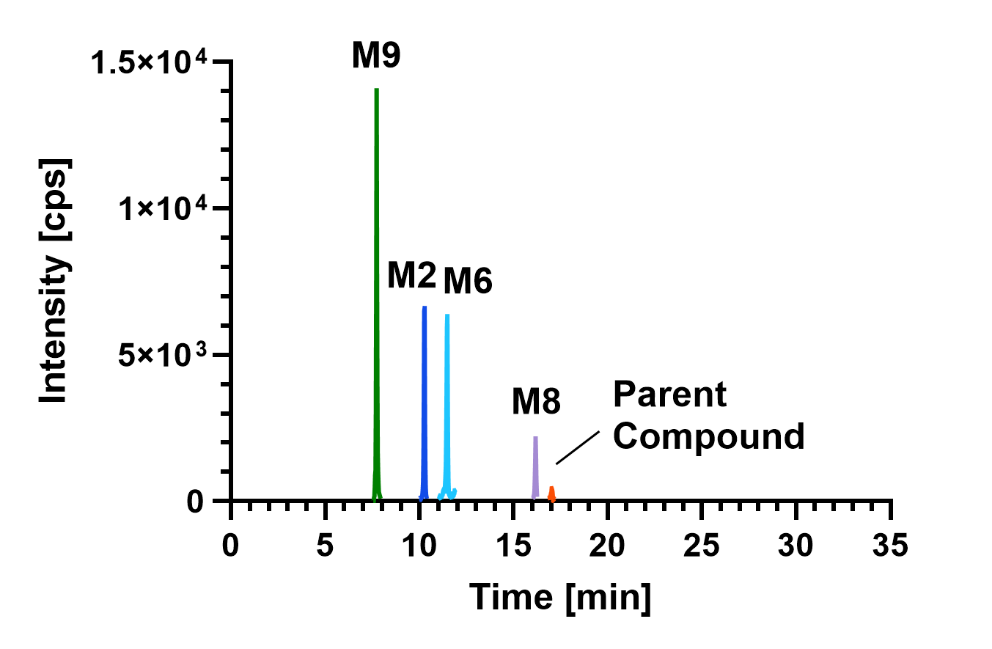


**Figure SI 3:** Extracted ion chromatogram (XIC) of the in vivo metabolites of N-pyrrolidino fluetonitazene in an authentic urine case sample.

***
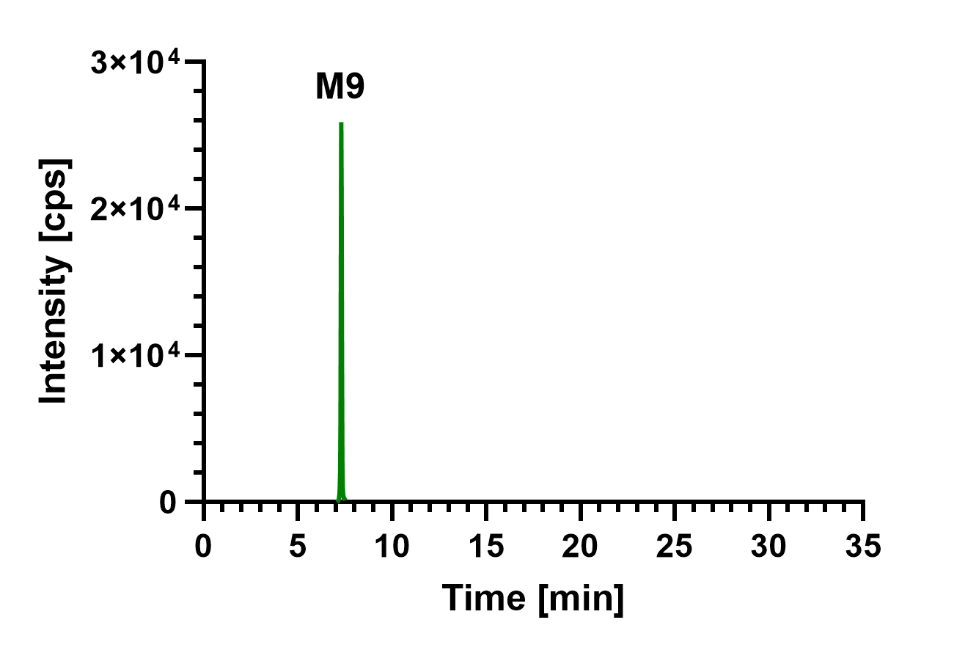
***

**Figure SI 4:** Extracted ion chromatogram (XIC) of the in vivo metabolites of N-pyrrolidino fluetonitazene in an authentic blood case sample.


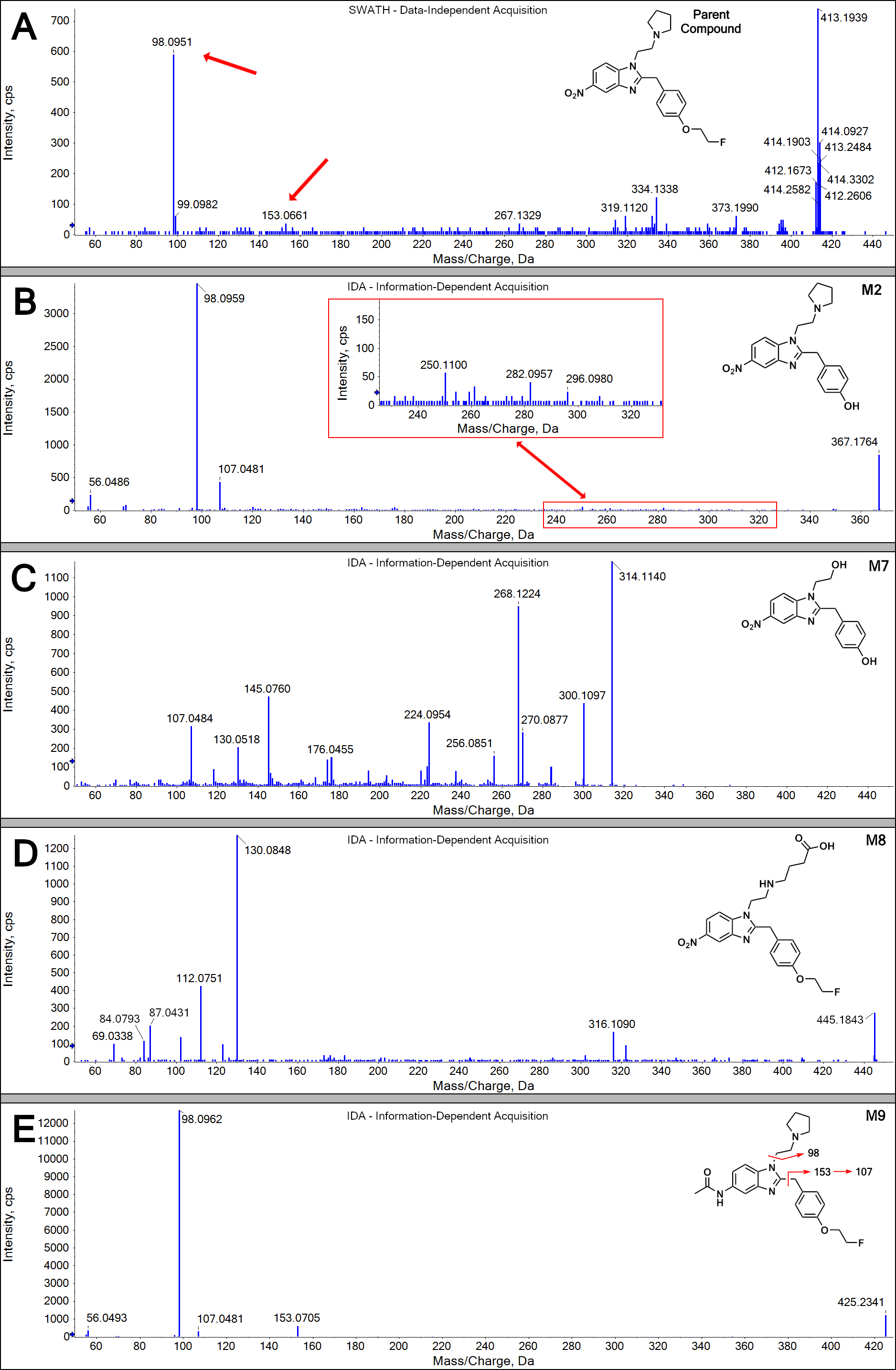


**Figure SI 5:** Data independent acquisition (SWATH) and information dependent acquisition (IDA) MS/MS spectra of (A) N-pyrrolidino fluetonitazene and (B-D) *in vivo* metabolites in a urine case sample and (E) in all three postmortem sample types. The parent compound underwent (B) O-dealkylation (**M2**), (C) O-dealkylation and hydrolytic deamination (**M7**) and (D) pyrrolidine ring opening via carboxylation (**M8**). (E) metabolite **M9** was formed by *N*-acetylation. Fragmentation of selected precursor ions was conducted with a CE of 35 eV and a CES of ± 15 eV.
